# Unraveling the Role of METTL3 in Helicobacter pylori-induced gastritis via m6A-CXCL1/NF-κB modulation

**DOI:** 10.1038/s41419-025-07841-4

**Published:** 2025-08-18

**Authors:** Qiutong Lu, Zhaopeng Wang, Shuixian Cao, Huan Wang, Nianshuang Li, Yi Hu, Wuhui Ding, Wei Zuo, Junbo Hong

**Affiliations:** 1 Department of Gastroenterology, Jiangxi Provincial Key Laboratory of Digestive Diseases, Jiangxi Clinical Research Center for Gastroenterology, Digestive Disease Hospital, The First Affiliated Hospital, Jiangxi Medical College, Nanchang University, Nanchang, Jiangxi PR China; 2 Department of Gastroenterology, Guixi People’s Hospital Guixi, Yingtan, Jiangxi PR China; 3 Department of Respiratory Medicine, The First Affiliated Hospital of Nanchang University, Nanchang, PR China

**Keywords:** Cell biology, Diseases

## Abstract

Helicobacter pylori (H. pylori) infection is a significant cause of gastric diseases, with its pathogenic mechanisms still not fully understood. This study investigates the role of METTL3, an enzyme involved in m6A methylation, in modulating the CXCL1/NF-κB signaling pathway in H. pylori-induced gastritis. Using both bioinformatics analysis of GEO database and experimental approaches including MeRIP, RIP assays, and immunostaining, this research highlights how METTL3 influences CXCL1 expression and NF-κB pathway activation. Results from both in vitro and in vivo models show that METTL3 increases inflammatory responses and apoptosis in gastric cells. Suppression of METTL3 resulted in decreased inflammation and apoptosis, suggesting its potential as a therapeutic target in gastritis management.

## Introduction

Helicobacter pylori (HP) infection is an etiological factor for global gastric inflammation, gastric ulcers, and gastric cancer. Extensive research has validated its direct correlation with the development of diverse gastric diseases [1–4]. HP infection could result in persistent inflammatory responses in the gastric mucosa, which could progress gradually to conditions such as gastric ulcers, gastric atrophy, intestinal epithelial dysplasia, and even gastric cancer [5–8]. However, the specific molecular mechanisms responsible for the inflammatory response in the gastric mucosa after H. pylori infection have not been fully elucidated.

In recent years, research has revealed that N6-methyladenosine (m6A) modification plays a crucial role in a range of physiological and pathological processes, including tumor progression, neurodevelopment, and cellular metabolism [9–11]. METTL3, as the primary methyltransferase of m6A, holds a crucial position in numerous cellular signal transduction pathways, and its aberrant expression is connected to the development of various diseases [12–14]. However, the specific role and underlying mechanism of METTL3 in the inflammatory response of the gastric mucosa following H. pylori infections are still not well understood.

CXCL1 is an established chemotactic factor that regulates the activation and migration of numerous inflammatory cells [15–17]. The expression of CXCL1 is regulated within the inflammatory environment of the gastric mucosa, and it is closely linked to the development and progression of inflammation [18]. The NF-κB signaling pathway is a central regulatory pathway involved in inflammatory reactions in various cells and tissues [19–21]. Recent studies suggest that METTL3 may play a role in regulating the m6A modification of CXCL1, which is involved in the inflammatory response of Gastritis and subsequently affects the NF-κB signaling pathway [22].

This study aimed to examine how METTL3 regulates the expression of CXCL1 via m6A modification. This process subsequently mediates the NF-κB signaling pathway and contributes to the molecular mechanism of gastric inflammation following H. pylori infection. Our objective is to contribute novel molecular targets and strategies for diagnosing, treating, and preventing Gastritis through comprehensive mechanism research. Simultaneously, we aim to enhance the precision of treatment plans in clinical medicine and mitigate the health risks associated with HP infection.

## Materials and methods

### Open the database to retrieve transcriptome sequencing data

Transcriptome RNA sequencing data for H. pylori (HPI) gastritis were retrieved from the Gene Expression Omnibus (GEO) database at http://www.ncbi.nlm.nih.gov/geo/. The sample information for the chip dataset is provided in Table S1. Ethical approval or informed consent was not required since the data were obtained from public databases.

### Differential gene analysis

The GEO dataset was utilized to select genes that met the criteria of |logFC | > 1 and P < 0.05 for filtering. The R language limma package was employed to identify differentially expressed genes. Heatmaps were generated using the R package “pheatmap”, while volcano plots were drawn using “ggplot2”. The analysis was conducted using R version 4.2.1, developed by the R Foundation for Statistical Computing [23].

### Construction of protein-protein interaction networks (PPI)

The STRING database, which integrates experimental data, text mining results from PubMed abstracts, and data from other databases, was utilized to investigate PPIs. The differentially expressed genes identified through the intersection of multiple gene sets were analyzed for PPI interactions within the human species, restricted to a confidence score of 0.7. The results were visualized using Cytoscape software [24].

### Gene function enrichment analysis

Gene Ontology (GO) and Kyoto Encyclopedia of Genes and Genomes (KEGG) enrichment analyses of the differentially expressed genes from the combined dataset were performed using the “clusterProfiler” package in R. Bar plots of the enrichment results for the three GO categories—biological process (BP), cellular component (CC), and molecular function (MF)—as well as the bar plot for KEGG enrichment analysis, were generated using the “enrichplot” package [25].

### Cell and HP cultivation, transfection, and grouping

GES-1 cells (FH0273, Fulenbio, China) were cultured in RPMI 1640 medium (11875101, Gibco, USA) supplemented with 10% fetal bovine serum (FBS) (12483020, Gibco, USA) and 1% penicillin/streptomycin (15070063, Gibco, USA) at 37 °C in a 5% CO_2_ humidified incubator [26].

Cell transfections were performed using Lipofectamine 2000 (11668019, Invitrogen, USA) with small interfering RNA negative control (si-NC), si-METTL3-1, si-METTL3-2, si-METTL3-3, si-CXCL1-1, si-CXCL1-2, and si-CXCL1-3. The siRNA sequences (Table S2) were designed using siDirect and synthesized by GenePharma (Shanghai, China). The silencing efficiency of si-METTL3 and si-CXCL1 sequences was assessed by RT-qPCR 48 hours post-transfection. For CXCL1 overexpression, CXCL1 cDNA was amplified by RT-PCR and cloned into the pcDNA3 vector. All expression plasmids were obtained from Shanghai Hanheng Biotechnology Co., Ltd. (Shanghai, China) at a concentration of 50 ng/ml. Helicobacter pylori (HP) (43504, ATCC, USA) was cultured in brain heart infusion (BHI) broth (237300, Gibco, USA) supplemented with 10% rabbit blood freeze-dried powder (MP20019-100 mL, Ocean, China) under microaerobic conditions at 37°C (10% CO_2_, 5% O_2_, 85% N_2_). Bacterial density was determined by measuring absorbance at 600 nm, with 1 OD600 = 1 × 10^9^ CFU/mL. Bacteria were resuspended in serum-free RPMI 1640 medium (11875101, Gibco, USA) for experimentation. GES-1 cells were treated with different concentrations of HP for 24 h and with 100 multiplicity of infection (MOI) of HP for various durations. Protein expression and mRNA levels of CXCL1 and METTL3 were assessed. Subsequently, GES-1 cells were uniformly treated with 100 MOI of HP for 24 h in subsequent experiments [27].

Lipopolysaccharides (LPS) (HY-D1056, MedChemexpress, USA) were used as NF-κB stimuli, and GES-1 cells were treated with LPS at 100 ng/mL for 24 h [28]. HPI GES-1 cells were divided into 10 groups for specific treatments (Table S3).

### Colony formation assay

GES-1 cells were pretreated with HP before being inoculated into a 6-well plate at a density of 500 cells per well. A single cell colony was allowed to proliferate for 14 days. The samples were then washed twice with phosphate-buffered saline (PBS) (10010023, Gibco, USA), fixed with 70% ethanol (E111989-500 ml, Aladdin, China) and stained with 0.5% crystal violet dye solution (C0121, Beyotime Biotechnology, China). The colonies were then counted, and the process was repeated three times for each group [29].

### ELISA

Cell culture supernatant was collected for ELISA. Levels of IL-6, IL-8, and TNFα in the supernatant were measured using a human IL-6 ELISA kit (ab100572, Abcam, UK), human IL-8 ELISA kit (ab214030, Abcam, UK), and human TNF-α ELISA kit (ab285312, Abcam, UK).

Mouse plasma was obtained by dissecting the eyeballs of mice that underwent experimental modeling for 9 weeks. The collected blood was allowed to stand for 30 min and then centrifuged at 3000 rpm for 10 min at 4 °C. The supernatant was used for ELISA analysis. CXCL1, IL-6, and TNFα in mouse serum were measured using the Mouse CXCL1 ELISA Kit (ab229426, Abcam, UK), Mouse IL-6 ELISA Kit (ab222503, Abcam, UK), and Mouse TNF alpha ELISA Kit (ab208348, Abcam, UK). Each experiment was repeated three times [30].

### Flow cytometry

Cell apoptosis was detected through the dual staining method of membrane-associated proteins V-FITC/PI (propidium iodide). GES-1 cells were inoculated at a density of 2 × 10^5^ cells per well in a 6-well plate and treated with trypsin (R001100, Gibco, USA). The cells were harvested into a 15 mL centrifuge tube and centrifuged at 800 g to collect the supernatant. The precipitate was resuspended in 500 μL of binding buffer and mixed with 5 μL of FITC and 5 μL of PI in the dark for 15 min, according to the apoptosis detection kit (556547, BD Biosciences, USA). After that, apoptosis analysis was performed using the BD FACSCalibur flow cytometer (BD Biosciences, USA). In this analysis, dead cells were represented by the upper left quadrant, late apoptotic cells by the upper right quadrant, early apoptotic cells by the lower right quadrant, and normal cells by the lower left quadrant. The apoptotic rate of cells was calculated as the sum of the percentages in the upper right and lower right quadrants [27]. Each experiment was repeated three times.

### Detecting biological membrane

HP suspension was incubated in a 48-well microfluidic plate to allow the formation of mature biofilms. After incubation, the waste culture medium was removed, and the biofilm was washed with a fresh culture medium to eliminate planktonic HP. The wells were rinsed with PBS to remove non-adherent cells and stained with a 1% (w/v) crystal violet solution for 15 min. The biofilm was observed using confocal laser scanning microscopy (CLSM, Olympus, Japan). Biofilms were also stained with the LIVE/DEAD BacLight Bacterial Viability Kit (L13152, Invitrogen, USA) in the dark for 15 min and then visualized using CLSM. Live cells were stained with SYTO 9 (excitation wavelength: 488 nm), while dead cells were stained with PI (excitation wavelength: 561 nm). Each experiment was repeated three times [31].

### Quantification of HP adherence using immunofluorescence staining

GES-1 cells were fixed at room temperature with 4% paraformaldehyde for 15 minutes and washed twice with PBS. The cells were permeabilized with 0.5% Triton X-100 (P0096, Beyotime Biotechnology, China) for 10 min. Afterward, the cells were incubated overnight at 4 °C with an anti-HP antibody (ab20459, 1:1000, Abcam, UK). Following three washes with PBS, the cells were incubated with an Alexa Fluor 488-conjugated secondary antibody (ab150077, 1:200, Abcam, UK) for 1 hour. After another three washes with PBS, DAPI staining (D3571, 10 μg/mL, Protein Tech, USA) was performed for 10 min. The samples were stored at 4 °C and examined under a fluorescence microscope (IMT-2, Olympus, Japan). Blue fluorescence indicated the GES-1 cell nuclei, while green fluorescence indicated HP [32]. The HP fluorescence area was quantified as a ratio to the DAPI fluorescence area using ImageJ software. For each cell sample, five slices were examined with 6–10 randomly chosen fields of view. Each experiment was repeated three times.

### Immunofluorescence staining

GES-1 cells and gastric mucosal tissues were fixed in 4% paraformaldehyde at room temperature for 15 min, followed by two washes with PBS. After that, they were permeabilized with 0.5% Triton X-100 (P0096, Beyotime Biotechnology, China) for 10 min. Next, the organizations and cells were incubated overnight at 4 °C with the following primary antibodies: METTL3 (ab195352, 1:100, Abcam, UK), CXCL1 (12335-1-AP, 1:100, Protein Tech, USA), NF-κB p65 (ab32536, 1:100, Abcam, UK), NF-κB p-p65 (ab131100, 1:100, Abcam, UK), GIF (K004862P, 1:100, Solarbio, China), Ki67 (ab15580, 1:200, Abcam, UK), MPO (ab208670, 1:200, Abcam, UK). After three washes with PBS, the sections were incubated with Alexa Fluor 647 (ab150083, 1:200, Abcam, UK) or Alexa Fluor 488-conjugated secondary antibodies (ab150077, 1:200, Abcam, UK) for 1 hour. Next, the sample was washed three times with a PBS solution. After that, it was stained with DAPI (D3571, 10 μg/mL, Abcam, UK) at room temperature for 10 min. The slices were stored at 4 °C and subsequently analyzed using a fluorescence microscope (IMT-2, Olympus, Japan). In the cell experiments, 5 slices were randomly examined in 6–10 fields per cell, with each experiment repeated 3 times. In the animal experiments, 6 mice were included in each group, and 5 slices from each mouse were randomly examined in 6–10 fields [33]. All the antibodies mentioned above, except CXCL1 (purchased from Protein Tech), were obtained from Abcam.

### Methylated RNA immunoprecipitation (MeRIP) assay

The binding sites of CXCL1 and m6A-related genes could be predicted using the SRAMP online database available at http://www.cuilab.cn/sramp. Reverse transcription was performed on 10 μl of m6A PolyA+ RNA from MeRIP using the iScript cDNA Synthesis Kit (1708890, Bio-Rad Laboratories, CA). Following a two-fold dilution of the cDNA, real-time quantitative PCR was performed utilizing primers sourced from ABI Prism 7900HT/FAST (1708890, Bio-Rad Laboratories, CA) and Integrated DNA Technologies, Inc. (Coralville, Iowa). The mRNA of MeRIP was quantitatively analyzed using the 2−ΔΔct method with non-immunoprecipitated input RNA. All primers used for real-time PCR are designed to encompass at least one intron. Amplification of a singular product is validated through visualization using agarose gel electrophoresis and/or melt curve analysis [34]. The primers used for MeRIP are displayed in Table S4. Furthermore, each experiment was repeated three times.

### RNA immunoprecipitation (RIP) assay

The RIP assay was performed using the RIP Kit (17-701, Millipore, USA) to detect the binding interactions between METTL3 and CXCL1, and BHLHE41 and CXCL1 proteins. When the GES-1 cells in a six-well plate reached 80-90% confluence, the culture medium was discarded, and the cells were washed with 1 mL of cold PBS. Equal volumes of RIPA lysis buffer (P0013B, Beyotime Biotechnology, China) were added, and the cells were incubated on ice for 5 min to induce lysis. The lysates were centrifuged at 1500 rpm for 10 min at 4 °C, and the supernatants were collected. A portion of the cell lysate was reserved as Input, while the remainder was incubated with specific antibodies for co-immunoprecipitation. For each co-precipitation reaction, 50 μL of lysate was used. Magnetic beads (17-701, Millipore, USA) were washed and resuspended in 100 μL of RIP Wash Buffer, followed by the addition of the appropriate antibody: rabbit anti-METTL3 (ab195352, 1:100, Abcam, USA), rabbit anti-BHLHE41 (1H18L6, 1:100, Thermo Fisher, USA), or rabbit anti-IgG (ab6715, 1:100, Abcam, USA). The mixture was incubated at room temperature for 30 min. The antibody-magnetic bead complexes were washed and resuspended in 900 μL of RIP Wash Buffer, and 100 μL of the cell lysate was added, incubating overnight at 4 °C. The samples were then placed on a magnetic stand to collect the magnetic bead-protein complexes. After digestion with proteinase K (17-701, Millipore, USA), RNA was extracted from both the samples and the Input for subsequent PCR detection of CXCL1 expression. Primer sequences are listed in Table S4 [35]. The experiment was repeated three times.

### Dual luciferase reporter gene experiment

The catRAPID online database (http://service.tartaglialab.com/) was used to predict the binding sites between CXCL1 and METTL3. The wild-type CXCL1 (CXCL1-WT) (5’-CTGGTAGCCGC-3’) and mutant CXCL1 (CXCL1-MUT) (5’-AGACCAUCGGCG-3’) were inserted into the pGL-3 luciferase reporter vector (4351372, Thermo Fisher, USA). The dual-luciferase reporter plasmids were transfected into GES-1 cells with either si-NC (5’-GATCGTACTCACATCCACACT-3’), si-METTL3 (5’-AGGAACAATCCATTGTTGAAAAA-3’), or si-BHLHE4 (5’-CCGCACAGATTAATAGAAATT-3’). After 48 h, the cells were collected and lysed, and the supernatant was centrifuged at 250 × g for 3–5 min. Luciferase activity was measured using the Dual-Luciferase® Reporter Assay System (E1910a, Promega, USA). Firefly luciferase activity was measured by adding 100 μL of Firefly luciferase working solution to the cell sample, and Renilla luciferase activity was measured by adding 100 μL of Renilla working solution. The relative luciferase activity was calculated as the ratio of Firefly luciferase to Renilla luciferase activity [36]. The experiment was repeated three times.

### RT-qPCR

Total RNA was extracted from cells and tissues using TRIzol (15596026, ThermoFisher, USA). The concentration and purity of the extracted total RNA were measured using a Nanodrop 2000 spectrophotometer (ThermoFisher, USA). RNA was reverse transcribed into cDNA using the PrimeScript RT reagent Kit (RR047A, Takara, Japan) following the manufacturer’s instructions. The synthesized cDNA was then subjected to RT-qPCR detection using the Fast SYBR Green PCR Kit (11736059, ThermoFisher, USA). Three replicates were performed for each well [37]. GAPDH was used as an internal reference. The relative expression was calculated using the 2^-ΔΔCt^ method. The experiment was repeated three times. The primer sequences utilized for the reverse transcription-quantitative polymerase chain reaction (RT-qPCR) in this study are presented in Table S5.

### Western blot

Cells were lysed using RIPA lysis buffer (P0013B, Beyotime Biotechnology, China). Protein concentration was quantified using the BCA protein assay kit (A53226, Thermo Fisher Scientific, USA). After separating proteins by polyacrylamide gel electrophoresis, they were transferred onto a PVDF membrane (PVH85R, Millipore, Germany) using the wet transfer method. The membrane was blocked with 5% BSA at room temperature for 1 hour and then incubated overnight at 4 °C with the following primary antibodies: rabbit anti-METTL3 (ab195352, 1:1000, Abcam, USA), rabbit anti-BHLHE41 (1H18L6, 1:100, Thermo Fisher, USA), rabbit anti-CXCL1 (12335-1-AP, 1:1000, Proteintech, USA), rabbit anti-p-IκBα (2859, 1:1000, Cell Signaling Technology, USA), rabbit anti-IκBα (4812, 1:1000, Cell Signaling Technology, USA), rabbit anti-NF-κB p65 (ab32536, 1:1000, Abcam, USA), rabbit anti-NF-κB p-p65 (ab131100, 1:1000, Abcam, USA), rabbit anti-GAPDH (ab181602, 1:1000, Abcam, USA), rabbit anti-cleaved caspase-3 (ab214430, 1:1000, Abcam, USA), rabbit anti-cleaved caspase-9 (9509, 1:1000, CST, USA), and rabbit anti-cleaved PARP (9541, 1:1000, CST, USA). After washing, the membrane was incubated with HRP-conjugated goat anti-rabbit IgG (ab6721, 1:5000, Abcam, UK) for 2 h. The membrane was washed three times with TBST for 5 min each, and proteins were detected using a chemiluminescent detection system. Protein quantification was performed using ImageJ 1.48 software (V1.48, National Institutes of Health, USA), and the grayscale values of the proteins were normalized to GAPDH or the corresponding non-phosphorylated protein [38]. The experiment was repeated three times. The antibodies for CXCL1 were obtained from Proteintech, while p-IκBα and IκBα antibodies were purchased from Cell Signaling Technology, and all other antibodies were sourced from Abcam.

### Constructing HPI-induced gastric injury mouse model

A total of 55 C57BL/6 mice (213, Charles River, China) were purchased, with body weights between 18 and 22 grams and ages between 6 and 8 weeks. The mice were housed in separate cages in an SPF-grade animal laboratory, with humidity maintained at 60%–65% and temperature at 22–25 °C. After a 1-week acclimation period, the experiment began, and the health of the mice was observed before the experiment.

Using CRISPR-Pro gene knockout technology, 40 METTL3^−/−^ mice and 20 CXCL1^−/−^ mice were generated. First, gRNA plasmids corresponding to the METTL3 target gene in mice (5’-GAGUUGAUUGAGGUAAAGCG-3’) and the CXCL1 target gene (5’-AUUGGCGAUAGGCGCCCCUA-3’) were designed and constructed. These plasmids were transcribed into RNA, and Cas9 mRNA was injected in vitro. Positive individuals from F0 heterozygous mice were screened and identified by sequencing. The F0 heterozygotes were then bred with WT mice to obtain F1 heterozygous mice, which were confirmed as PCR-positive and identified by sequencing. F1 mice of the same genotype from the same F0 mouse were selected, and after reaching sexual maturity, they were paired to produce F2 mice. F2 METTL3^−/−^ mice were confirmed by PCR testing and sequencing. Additionally, 12 METTL3^−/−^ mice were successfully generated from the same cage as WT mice, with a success rate of 30% [39].

To construct the HPI gastritis mouse model, 24 mice were divided into the following 4 groups: WT group (uninfected HP mice), HPI group (HPI gastritis mice), METTL3^−/−^ group (uninfected METTL3^−/−^ mice), HPI + METTL3^−/−^ group (HPI gastritis METTL3^−/−^ mice), CXCL1^−/−^ group (uninfected CXCL1^−/−^ mice), and HPI + CXCL1^−/−^ group (HPI gastritis CXCL1^−/−^ mice), with 6 mice in each group. Each group of mice received daily injections of 200 μL of an antibiotic cocktail for 7 consecutive days to eliminate most of the remaining intestinal pathogens and reduce contamination. Six mice were randomly assigned to each group. Every other day, 3 × 10^8^ CFU HP (0.4 mL of saline) was administered via oral gavage, twice a day, and completed within 1 h [27]. The experimental protocol and animal use procedures were approved by the Institutional Animal Ethics Committee.

### Rapid urease test

The fresh mouse gastric mucosal tissue was added to the reagent tube using a rapid urease test kit (Shandong Bomaida Biotechnology Co., Ltd, China), produced on July 29, 2019. The color change inside the tube was observed within 30 min. If the solution changes from yellow to red, it indicates a positive test result, suggesting the presence of HPI in the biopsy tissue. If the solution remains yellow, it indicates a negative test result, suggesting the absence of HPI [40].

### H&E staining

The H&E staining was performed using the H&E Staining Kit (C0105, Beyotime, China) with the following steps: (1) Mouse gastric mucosal tissues were fixed in 10% neutral buffered formalin at 4 °C for 24 h. (2) The tissues were dehydrated, embedded in paraffin, and sectioned. (3) The sections were deparaffinized with xylene, rehydrated through a graded alcohol series, and rinsed with distilled water. (4) Hematoxylin staining solution was applied for 5–10 min. (5) The sections were rinsed with deionized water for approximately 10 min to remove excess stains. (6) Eosin staining solution was applied for 30 s to 2 min. (7) The sections were dehydrated again through a graded alcohol series and cleared with xylene. (8) Finally, the slides were mounted with neutral resin or another mounting medium, and observed under an inverted microscope (IX73, OLYMPUS, Japan) for imaging. Based on literature standards and the criteria in Table S6, two pathologists independently evaluated the slides using a double-blind method, assigning scores for inflammation from anatomical (CORPUS) and pathological (ANTRUM) perspectives. Inflammation was scored as follows: 0 for no inflammation, 1 for mild, 2 for moderate, and 3 for significant inflammation. The overall inflammation grade was determined as normal (STAGE 0) to significant (STAGE III), and an average score was calculated [41].

### TUNEL staining

Cell apoptosis in mouse gastric mucosal tissue was detected using the TUNEL Kit (C1098, Beyotime, China). The slices were treated with 3% H2O2 and added 50 μL of TUNEL culture medium. Subsequently, the slices were incubated at 37 °C for 60 min in a dark environment. Next, add 50 μL of the streptavidin-HRP working solution and incubate it for 30 min. Apply 0.2–0.5 mL of the suitable DAB staining solution to the section and let it stand for 5 min, utilizing an appropriately sized organizational block. After the staining process, five slices were randomly selected from each of the six mice. These slices were observed using an inverted microscope (IX73, Olympus, Japan). For each mouse, 6–10 fields were examined, and the count of positive cells was recorded. Next, determine the total number of cells and count the number of apoptotic cells. Subsequently, calculate the apoptosis rate in the cell population [42].

### Immunohistochemistry experiment

The gastric mucosal sample is fixed using a 4% paraformaldehyde solution, dehydrated, clarified, embedded in paraffin, and subsequently sliced. In immunohistochemical staining, the slides were initially dewaxed and rehydrated, followed by antigen retrieval through immersion in PBS and subsequent boiling of the liquid using a microwave oven. Following that, staining was conducted using the universal two-step method with the PV-9000 kit (Protein Tech, USA) in accordance with the instructions provided by the manufacturer. The staining results were observed and recorded using the CX43 optical microscope from OLYMPUS, Japan. Positive cells manifest as a light brown or tan staining outcome. Five slices were randomly selected from each of the six mice. Six to ten fields were observed, and positive cells were recorded. The analysis was carried out using the image analysis system, namely the Aperio Scanscope System (Vista, CA) [43].

### Statistical analysis

Statistical analysis for this study was performed using IBM SPSS Statistics software (version 21.0, USA). Quantitative data is represented by mean ± standard deviation. To begin with, it is recommended to conduct normality and homogeneity of variances tests. If the data meets the assumptions of normal distribution and homogeneity of variances, the independent t-test could be employed to compare the two groups. In the case of repeated measurements, one-way ANOVA or repeated measures ANOVA was utilized to compare multiple groups. Pearson correlation analysis is employed to examine the correlation among indicators. A *P*-value lower than 0.05 indicates statistical significance [44].

## Results

### Bioinformatics analysis indicates that high expression of CXCL1 may promote the occurrence and development of HPI gastritis

HP is a human pathogen and a primary contributor to the development of pathological damage, ranging from chronic Gastritis to gastric cancer [45]. Gastritis and HP infection are linked to persistent colonization of the gastric mucosa. Most severe gastric diseases develop due to chronic mucosal inflammation, which is mediated by the secretion of pro-inflammatory cytokines and chemokines, mainly IL-8, triggered by activated neutrophils and macrophages [46]. Nevertheless, the precise mechanism by which HP infection causes Gastritis remains uncertain. Thus, we investigated the analysis of HPI gastritis by retrieving transcriptome RNA sequencing data from the GEO database. Genes with an absolute log-fold change (|logFC | ) greater than 1 and a *p*-value (P) less than 0.05 were filtered as differentially expressed genes. In the GSE5081 dataset, 347 genes were upregulated, and 375 genes were downregulated (Fig. S1A). In the GSE60427 dataset, 1032 genes were upregulated, and 299 were downregulated (Fig. S1B). In the GSE6624 dataset, 1103 genes were upregulated, and 452 genes were downregulated (Fig. S1C).

We intersected the differentially expressed genes of HPI gastritis from the three datasets with the gene set of HP from the GeneCards database. First, we took the intersection of the upregulated genes across the four datasets and identified 22 common differentially expressed genes (CCL20, CD40LG, CHI3L1, CLEC4A, CXCL1, CXCL13, CXCL2, CXCL3, CXCL5, CXCL9, FGR, GPR65, HLA-DOB, ITGB7, LCN2, LTF, MS4A1, PDE4B, S100A8, S100A9, SERPINA3, TNFRSF4) (Fig. S2A). For the downregulated genes, one common differentially expressed gene (CCKAR) was identified by taking the intersection of the four datasets (Fig. S2B). The heatmap of the expression of these 23 common differentially expressed genes across the datasets is shown in (Fig. S3A–C).

To further filter the differentially expressed genes of HPI gastritis, we input these 23 common genes into the STRING database, specifying the species as human and setting the confidence score at 0.7. This allowed us to obtain the PPI network of the proteins encoded by the differentially expressed genes (Fig. S2C). From the PPI network, we observed that CXCL1 had the highest degree at 12. Additionally, high expression of CXCL1 in the Gastritis group was observed in all three HPI gastritis datasets (Fig. S2D–I). According to the literature, CXCL1 is one of the most important chemokines involved in the development of various inflammatory diseases, and its expression is elevated during the inflammatory response [47]. Based on these results, we speculate that the increased expression of CXCL1 may play a role in promoting the progression of HPI gastritis.

To further demonstrate the role of CXCL1 in promoting HPI gastritis, we divided the gastritis samples from the three datasets into a CXCL1 low-expression (CXCL1-Low) group and a CXCL1 high-expression (CXCL1-High) group based on CXCL1 expression levels, and performed differential analysis. In the GSE5081 dataset, 381 upregulated genes and 358 downregulated genes were identified (Fig. S1D); in the GSE60427 dataset, 160 upregulated genes and 68 downregulated genes were identified (Fig. S1E); in the GSE60662 dataset, 415 upregulated genes and 431 downregulated genes were identified (Fig. S1F).

The analysis suggests that CXCL1 promotes the progression of HPI gastritis by regulating signaling pathways such as the TNF, NF-κB, and chemokine signaling pathway.

### Potential regulatory role of METTL3 in m6A modification of CXCL1 in HPI gastritis

m6A is the most prevalent post-transcriptional modification in both mRNA and non-coding RNA. This modification plays a crucial role in determining the fate of RNA, including processes such as splicing, localization, stability, translation efficiency, and nuclear export [48–50]. Currently, most research on the role of m6A in HPI-related diseases concentrates on gastric cancer, with comparatively less emphasis on the role of m6A RNA modification in HPI-related non-cancerous diseases. Literature reports suggest that HPI increases levels of gastric inflammation, both extracellularly and intracellularly, through m6A modification [51].

**Fig. 1:**
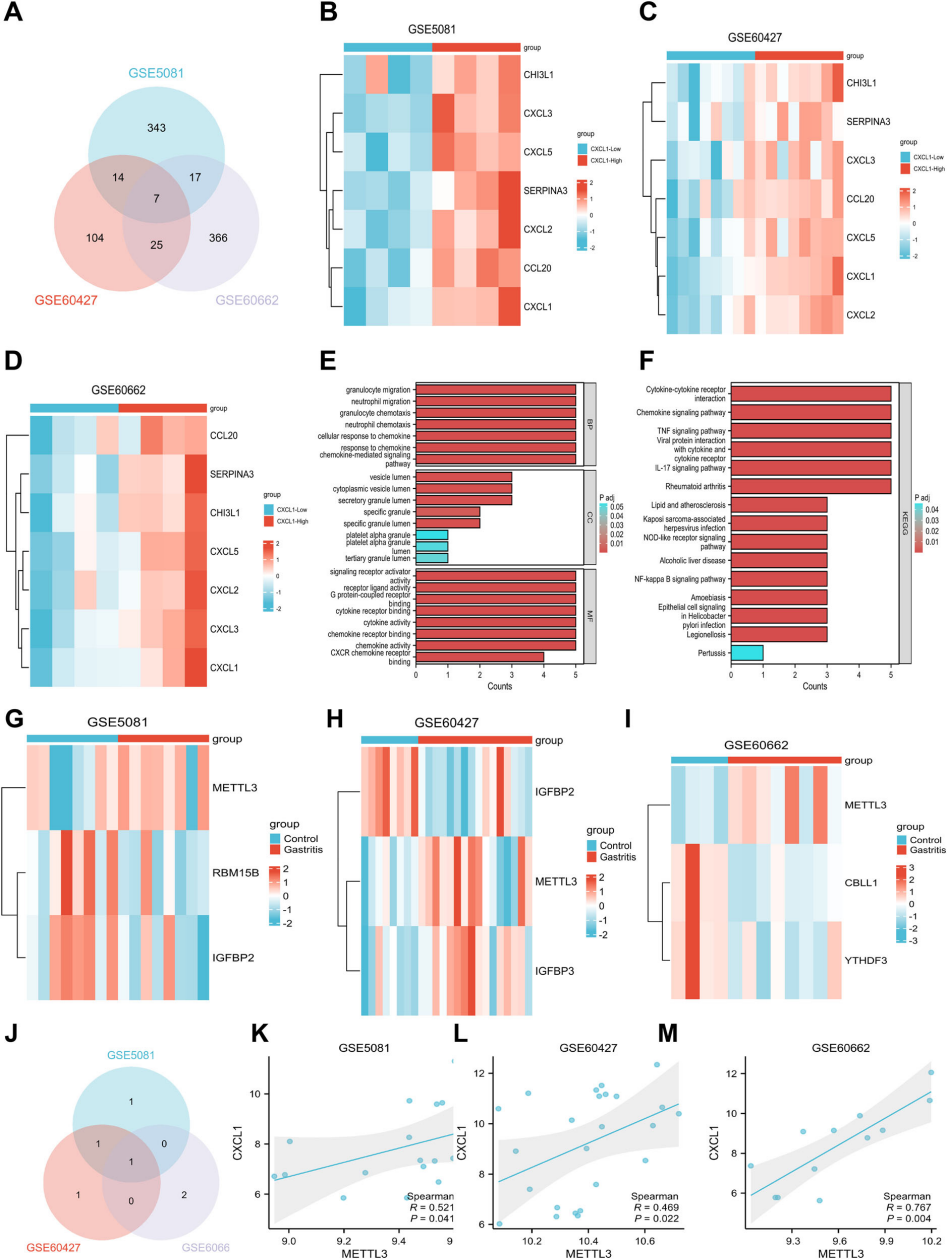
Functional and differential gene analysis of CXCL1 and m6A modifications in gastric tissue. **A** Venn diagram of the intersection of differentially expressed genes from three HPI gastritis datasets. **B** Heatmap of the expression of the 7 intersecting differentially expressed genes in the GSE5081 dataset (CXCL1-Low group: n = 4; CXCL1-High group: n = 4). **C** Heatmap of the expression of the 7 intersecting genes in the GSE60427 dataset (CXCL1-Low group: n = 8; CXCL1-High group: n = 8). **D** Heatmap of the expression of the 7 intersecting genes in the GSE60662 dataset (CXCL1-Low group: n = 4; CXCL1-High group: n = 4). **E** GO functional analysis of the 7 intersecting differentially expressed genes in terms of biological process (BP), cellular component (CC), and molecular function (MF). **F** KEGG pathway enrichment analysis of the 7 intersecting differentially expressed genes. **G** Heatmap of the expression of 3 differentially expressed m6A modification-related genes in the GSE5081 dataset (Control group: n = 8; Gastritis group: *n* = 8). **H** Heatmap of the expression of 3 differentially expressed m6A modification-related genes in the GSE60427 dataset (Control group: n = 8; Gastritis group: n = 16). **I** Heatmap of the expression of 3 differentially expressed m6A modification-related genes in the GSE60662 dataset (Control group: n = 4; Gastritis group: n = 8). **J** Venn diagram of the intersection of differential genes related to m6A modification in three HPI gastritis datasets. **K** Correlation analysis of METTL3 and CXCL1 in the GSE5081 dataset. **L** Correlation analysis of METTL3 and CXCL1 in the GSE60427 dataset. **M** Correlation analysis of METTL3 and CXCL1 in the GSE60662 dataset. Values are expressed as mean ± standard deviation. *P < 0.05, **P < 0.01, ***P < 0.001 compared to Control.

By intersecting the differentially expressed genes from these three datasets, we identified seven common differentially expressed genes (Fig. 1A). The expression patterns of these seven common differentially expressed genes in each dataset are shown in (Fig. 1B–D). To explore the function of CXCL1 in HPI gastritis, we conducted GO functional analysis and KEGG pathway analysis on the common differentially expressed genes from the three datasets.

The results of GO functional analysis showed that the differentially expressed genes were mainly enriched in biological processes (BP) such as chemokine-mediated signaling pathways, and neutrophil chemotaxis. In terms of cellular components (CC), the differentially expressed genes were primarily enriched in terms such as secretory granule lumen, cytoplasmic vesicle lumen, vesicle lumen, and specific granule lumen. Regarding molecular functions (MF), the genes were mainly enriched in chemokine activity, chemokine receptor binding, and cytokine activity (Fig. 1E). KEGG pathway analysis indicated that the candidate genes were primarily enriched in pathways such as the IL-17 signaling pathway, TNF signaling pathway, chemokine signaling pathway, cytokine-cytokine receptor interaction, and the NF-κB signaling pathway (Fig. 1F). To examine the overall expression of genes related to m6A modification in patients with HPI gastritis, we extracted m6A-related genes from three datasets and conducted a differential analysis. The differential genes in the GSE5081 dataset are METTL3, RBM15B, and IGFBP2 (Fig. 1G, Fig. S3D). In the GSE60427 dataset, the differential genes are IGFBP2, METTL3, and IGFBP3 (Fig. 1H, Fig. S3E). Additionally, in the GSE60662 dataset, the differential genes include METTL3, CBLL1, and YTHDF3 (Fig. 1I, Fig. S3F). The m6A-related differentially expressed genes were intersected from these three datasets, and subsequently, the METTL3 gene was filtered out (Fig. 1J). According to the existing literature, METTL3 has been shown to promote the growth and metastasis of gastric cancer cells [52]. However, research on m6A modification in HPI gastritis, a non-cancerous disease, is still limited.

According to literature studies, it has been discovered that YTHDF1 can increase the expression of CXCL1 by enhancing the translation of p65. This study suggests the potential involvement of m6A-related genes in the methylation of CXCL1 [53]. To evaluate the existence of a regulatory relationship between METTL3 and CXCL1, we conducted correlation analysis on their expressions in three GEO datasets. The results demonstrated a positive correlation between METTL3 and CXCL1 in all three datasets (Fig. 1K–M). We plan to investigate further to determine whether METTL3 could regulate the m6A modification of CXCL1 in HPI gastritis based on the above results.

### METTL3-mediated m6A modification of CXCL1 drives inflammation and apoptosis in HPI-treated GES-1 Cells

**Fig. 2:**
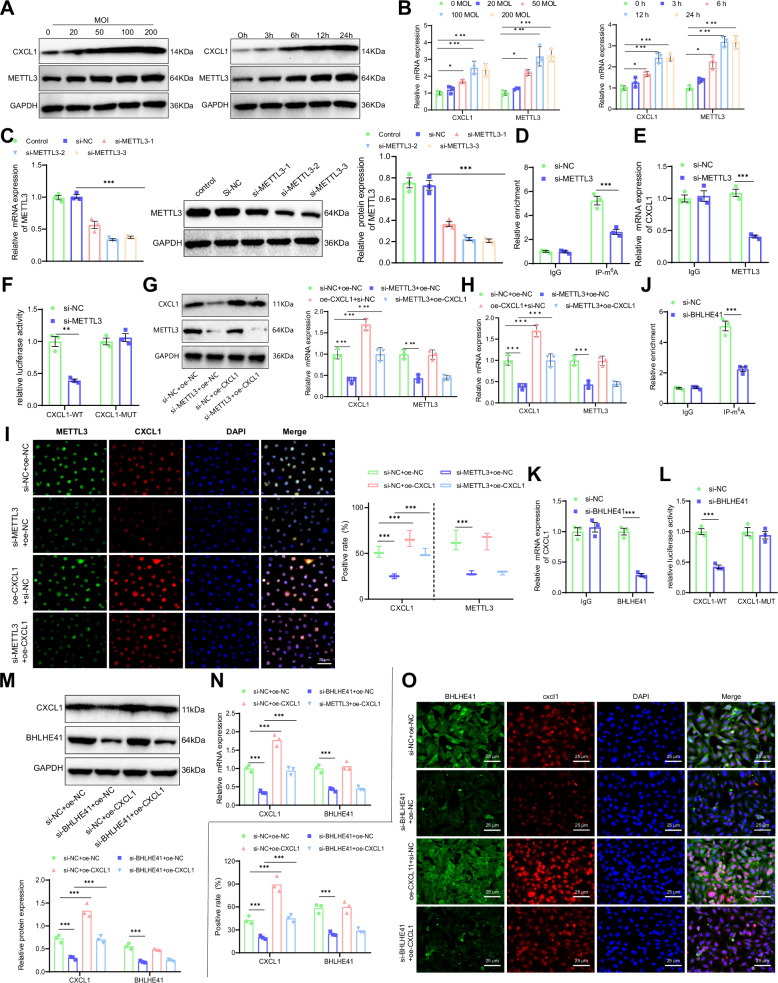
Validation of METTL3-CXCL1 Target Relationship. **A** Western blot analysis of CXCL1 and METTL3 protein levels in GES-1 cells after co-culture with HP at different MOIs (0, 20, 50, 100, and 200) and for different durations (0, 3, 6, 12, and 24 h). **B** RT-qPCR analysis of CXCL1 and METTL3 mRNA levels in GES-1 cells after co-culture with HP at different MOIs (0, 20, 50, 100, and 200) and for different durations (0, 3, 6, 12, and 24 h). **C** RT-qPCR and Western blot screening of three si-METTL3 sequences. **D** MeRIP-qPCR experiment detecting m6A methylation modifications of CXCL1 mRNA in GES-1 cells. **E** RIP-qPCR experiment detecting the target relationship between CXCL1 mRNA and METTL3 in GES-1 cells. **F** Dual-luciferase reporter assay detecting the target relationship between CXCL1 and METTL3 in GES-1 cells. **G** Western blot analysis of CXCL1 and METTL3 protein expression levels in HP-infected GES-1 cells. **H** RT-qPCR analysis of CXCL1 and METTL3 mRNA levels in HP-infected GES-1 cells. **I** Immunofluorescence staining detecting the expression levels of CXCL1 and METTL3 in HP-infected GES-1 cells. **J** MeRIP-qPCR experiment detecting m6A methylation modifications of CXCL1 mRNA in GES-1 cells. **K** RIP-qPCR experiment detecting the target relationship between CXCL1 mRNA and BHLHE41 in GES-1 cells. **L** Dual-luciferase reporter assay detecting the target relationship between CXCL1 and BHLHE41 in GES-1 cells. **M** Western blot analysis of CXCL1 and BHLHE41 protein expression levels in HP-infected GES-1 cells. **N** RT-qPCR analysis of CXCL1 and BHLHE41 mRNA levels in HP-infected GES-1 cells. **O** Immunofluorescence staining detecting the expression levels of CXCL1 and BHLHE41 in HP-infected GES-1 cells. Scale bar: 25 μm. Values are presented as the mean ± standard deviation, and all cell experiments were repeated three times. ***indicates *P* < 0.001.

In order to investigate the impact of HP on the expression of METTL3 and CXCL1, we co-cultured HP with human gastric epithelial cells (GES-1) at varying multiplicities of infection (MOI) for 24 h. Moreover, GES-1 cells were co-cultured with HP at an MOI of 100 for different durations to assess the effects on METTL3 and CXCL1 expression. We detected the expression levels of CXCL1 and METTL3. Western blot and RT-qPCR analysis revealed that following HP treatment, the expression of CXCL1 in GES-1 cells progressively increased, showing a correlation with both the multiplicity of infection (MOI) and exposure duration (Fig. 2A, B). In summary, HP has the ability to enhance the expression of CXCL1 and METTL3 in GES-1 cells. Subsequent experiments involved the intervention of GES-1 cells with HP at an MOI of 100 for 24 hours.

To investigate the regulatory relationship between CXCL1 and METTL3, we conducted three si-METTL3 transfections in GES-1 cells. The results of RT-qPCR demonstrated that si-METTL3-2 had the most effective silencing efficiency. As a result, si-METTL3-2 (si-METTL3) was selected for subsequent experiments (Fig. 2C). Subsequently, the results of the MeRIP experiment demonstrated a reduction in the m6A modification level of CXCL1 mRNA in the si-METTL3 group compared to the si-NC group (Fig. 2D). Moreover, the RIP experiment results showed a substantial decrease in the levels of CXCL1 mRNA pulled down by METTL3 in the si-METTL3 group compared to the si-NC group (Fig. 2E). Additionally, the findings of the dual-luciferase reporter gene experiment indicated a marked reduction in dual-luciferase activity in the si-METTL3 group compared to the si-NC group in the WT (wild-type) CXCL1 group, while no change was observed in the MUT (CXCL1 mutant) group, where the CXCL1 gene had been mutated to prevent METTL3 binding (Fig. 2F). The aforementioned results indicate that METTL3 selectively regulates m6A modification on CXCL1 mRNA.

Western blot and RT-qPCR detection results revealed that the protein and mRNA levels of CXCL1 and METTL3 were downregulated in the si-METTL3+oe-NC group compared to the si-NC+oe-NC group. There was no change in the METTL3 protein and mRNA levels in the oe-CXCL1+si-NC group, but there was an upregulation in the protein and mRNA levels of CXCL1. Moreover, the protein and mRNA levels of CXCL1 were upregulated in the si-METTL3+oe-CXCL1 group compared to the si-METTL3+oe-NC group (Fig. 2G, H). The immunofluorescence staining results are consistent with the results obtained from the Western blot experiment (Fig. 2I). In summary, HP induces METTL3 to perform m6A modification on CXCL1, thereby influencing its expression.

METTL3, as a potential therapeutic target for CRC immunotherapy, can reverse immunosuppression through the m6A-BHLHE41-CXCL1 axis. The inhibition of METTL3, combined with anti-PD1 therapy, has shown promising anti-tumor effects in CRC [22]. To investigate whether BHLHE41 is involved in the regulation of CXCL1 expression mediated by METTL3 in HP-treated GES-1 cells, we designed relevant experiments for validation. Through MeRIP experiments, it was found that the m6A modification level of CXCL1 mRNA in the si-BHLHE41 group was significantly reduced (Fig. 2J); RIP experiment results showed that the level of CXCL1 mRNA pulled down by BHLHE41 was significantly decreased in the si-BHLHE41 group (Fig. 2K); dual-luciferase reporter assay results showed that the luciferase activity in the si-BHLHE41 group was significantly reduced, while no significant change was observed in the MUT group (Fig. 2L). These results suggest that BHLHE41 targets and regulates the m6A modification of CXCL1 mRNA. Western blot and RT-qPCR results showed that the protein and mRNA levels of CXCL1 and BHLHE41 in the si-BHLHE41+oe-NC group were significantly downregulated, while the BHLHE41 protein and mRNA levels in the oe-CXCL1+si-NC group did not show significant changes, but the CXCL1 protein and mRNA levels were significantly upregulated. Compared with the si-BHLHE41+oe-NC group, the CXCL1 protein and mRNA levels in the si-BHLHE41+oe-CXCL1 group were significantly upregulated (Fig. 2M, N). Immunofluorescence staining results were consistent with the Western blot results (Fig. 2O). In conclusion, BHLHE41 affects the expression of CXCL1 by regulating its m6A modification.

**Fig. 3:**
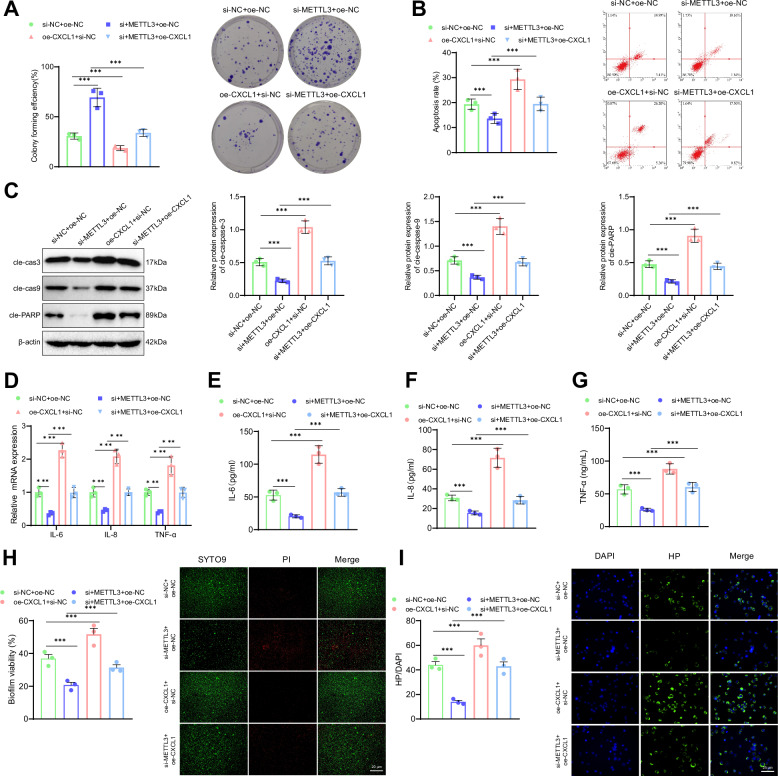
Impact of METTL3/CXCL1 Signaling Axis on HPI GES-1 Cells. **A** Colony formation assay to assess cell viability in HPI GES-1 cells. **B** Flow cytometry analysis to measure cell apoptosis in HPI GES-1 cells. **C** Western blot analysis of cleaved caspase-3, cleaved caspase-9, and cleaved PARP levels. **D** mRNA levels of IL-6, IL-8, and TNF-α in HPI GES-1 cells as detected by RT-qPCR. **E** IL-6 content in HPI GES-1 cells as measured by ELISA. **F** IL-8 content in HPI GES-1 cells as measured by ELISA. **G** TNF-α content in HPI GES-1 cells as measured by ELISA. **H** HP biofilm formation in HPI GES-1 cells as detected by immunofluorescence staining; Scale bar: 50 μm. **I** Adhesion of HP in HPI GES-1 cells as detected by immunofluorescence staining; Scale bar: 25 μm. Values are expressed as mean ± standard deviation. ***P < 0.001.

Subsequently, we will explore the impact of the METTL3/CXCL1 signaling pathway on the phenotype of GES-1 cells treated with HPI. First, the experimental results demonstrated increased cell cloneability in the si-METTL3+oe-NC group compared to the si-NC+oe-NC group. Conversely, cell cloneability showed a notable decrease in the oe-CXCL1+si-NC group. Additionally, the si-METTL3+oe-CXCL1 group exhibited a decrease in cell cloneability compared to the si-METTL3+oe-NC group (Fig. 3A). The results of the subsequent flow cytometry assay revealed that the apoptosis rate of cells was reduced in the si-METTL3+oe-NC group compared to the si-NC+oe-NC group.

Conversely, the apoptosis rate of cells in the oe-CXCL1+si-NC group was increased. Furthermore, the apoptosis rate of cells in the si-METTL3+oe-CXCL1 group was higher compared to the si-METTL3+oe-NC group (Fig. 3B). Meanwhile, WB results showed that compared with the si-NC+oe-NC group, the levels of cleaved caspase-3, cleaved caspase-9, and cleaved PARP in the si-METTL3+oe-NC group were decreased, while the levels of cleaved caspase-3, cleaved caspase-9, and cleaved PARP in the CXCL1+si-NC group were increased; compared with the si-METTL3+oe-NC group, the levels of cleaved caspase-3, cleaved caspase-9, and cleaved PARP in the si-METTL3+oe-CXCL1 group were increased (Fig. 3C). Subsequent RT-qPCR and ELISA results showed that compared with the si-NC+oe-NC group, the mRNA expression levels and content of IL-6, IL-8, and TNF-α in the si-METTL3+oe-NC group were significantly decreased, while the mRNA expression levels and content of IL-6, IL-8, and TNF-α in the oe-CXCL1+si-NC group were significantly increased; compared with the si-METTL3+oe-NC group, the mRNA expression levels and content of IL-6, IL-8, and TNF-α in the si-METTL3+oe-CXCL1 group were significantly increased (Fig. 3D–G).

Finally, the immunofluorescence staining results indicated that in comparison to the si-NC+oe-NC group, the green fluorescence was attenuated, and the biofilm was impaired in the si-METTL3+oe-NC group. Conversely, the green fluorescence was intensified, leading to the formation of a mature biofilm in the oe-CXCL1+si-NC group. Furthermore, the biofilm formation increased in the si-METTL3+oe-CXCL1 group compared to the si-METTL3+oe-NC group (Fig. 3H). Immunofluorescence staining was performed to evaluate HP adhesion. The results showed that in the si-METTL3+oe-NC group, compared to the si-NC+oe-NC group, the green fluorescence was reduced, and HP adhesion was decreased in the cells. Conversely, in the oe-CXCL1+si-NC group, the green fluorescence was enhanced, and HP adhesion was increased. Moreover, in the si-METTL3+oe-CXCL1 group, HP adhesion was further increased compared to the si-METTL3+oe-NC group (Fig. 3I).

In conclusion, METTL3 enhances the expression of CXCL1 by regulating the m6A modification of RNA. This regulation exacerbates cell apoptosis, biofilm formation, HP adhesion, and the inflammatory response induced by HPI treatment.

### CXCL1 regulates the NF-κB signaling pathway, promoting apoptosis, biofilm formation, and inflammatory responses in HP1-treated GES-1 cells

Our analysis of GO and KEGG indicated an association between the upregulation of CXCL1 in HPI and the NF-κB pathway, as demonstrated in Fig. 1F. Hence, we postulate that HPI could potentially activate the NF-κB pathway by upregulating CXCL1 expression. LPS activates the NF-κB signaling pathway by binding to Toll-like receptors (TLRs) on the cell surface. Hence, we selected LPS as the agonist to activate the NF-κB signaling pathway [54].

**Fig. 4:**
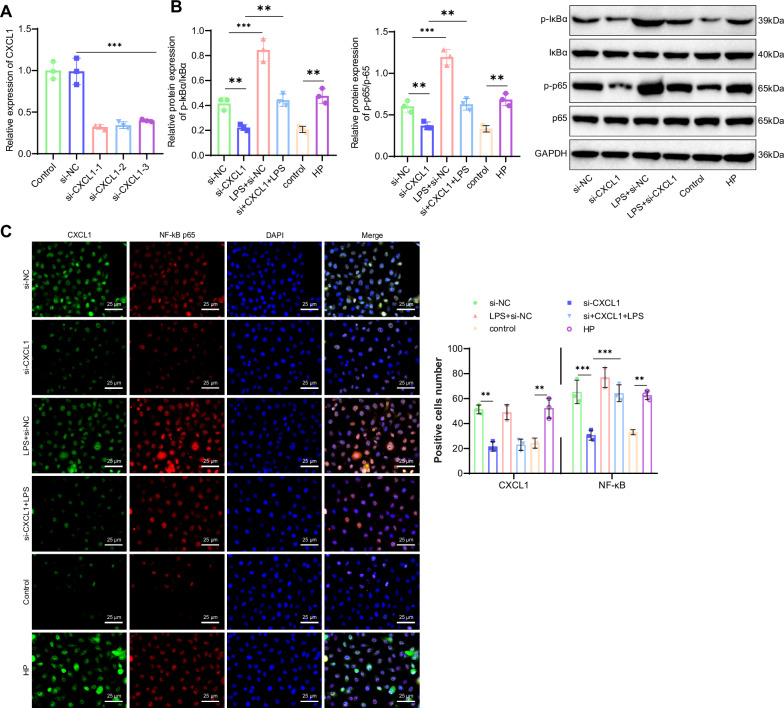
Validation of the relationship between CXCL1 and the NF-κB signaling pathway. **A** RT-qPCR screening of three si-CXCL1 sequences. **B** Western blot analysis of p-IκBα, IκBα, p-p65, and p65 protein expression levels in HPI-treated GES-1 cells. The bar charts represent the ratios of p-IκBα/IκBα and p-p65/p65 protein levels. **C** Immunofluorescence staining of CXCL1 and NF-κB expression levels in HPI-treated GES-1 cells, scale bar: 25 μm. Values are presented as the mean ± standard deviation, and all cell experiments were repeated three times. *** indicates *P* < 0.001.

First, GES-1 cells were transfected with three si-CXCL1 sequences, and RT-qPCR results showed that si-CXCL1-1 had the highest silencing efficiency. Therefore, si-CXCL1-1 (CXCL1-1) was selected for subsequent experiments (Fig. 4A). Western blot results showed that, compared to the Control group, the protein levels of the p-IκBα/IκBα and p-p65/p65 ratios were significantly upregulated in the HP group. Compared to the si-NC group, the p-IκBα/IκBα and p-p65/p65 protein levels were significantly downregulated in the si-CXCL1 group, while in the LPS+si-NC group, the p-IκBα/IκBα and p-p65/p65 protein levels were significantly upregulated. Compared to the si-CXCL1 group, the protein levels of p-IκBα/IκBα and p-p65/p65 were significantly upregulated in the si-CXCL1 + LPS group (Fig. 4B). Immunofluorescence staining results showed that, compared to the Control group, the fluorescence intensity of CXCL1 and p-p65 was enhanced in the HP group. Compared to the si-NC group, the fluorescence intensity of CXCL1 and p-p65 was decreased in the si-CXCL1 group, while in the LPS+si-NC group, the fluorescence intensity of CXCL1 and p-p65 was enhanced. Compared to the si-CXCL1 group, the fluorescence intensity of CXCL1 and p-p65 was enhanced in the si-CXCL1 + LPS group (Fig. 4C). These results indicate that si-CXCL1 can inhibit the nuclear translocation of p65, thereby suppressing the NF-κB signaling pathway, which is corroborated by the Western blot results. Thus, CXCL1 is an upstream regulator of the NF-κB signaling pathway.

**Fig. 5:**
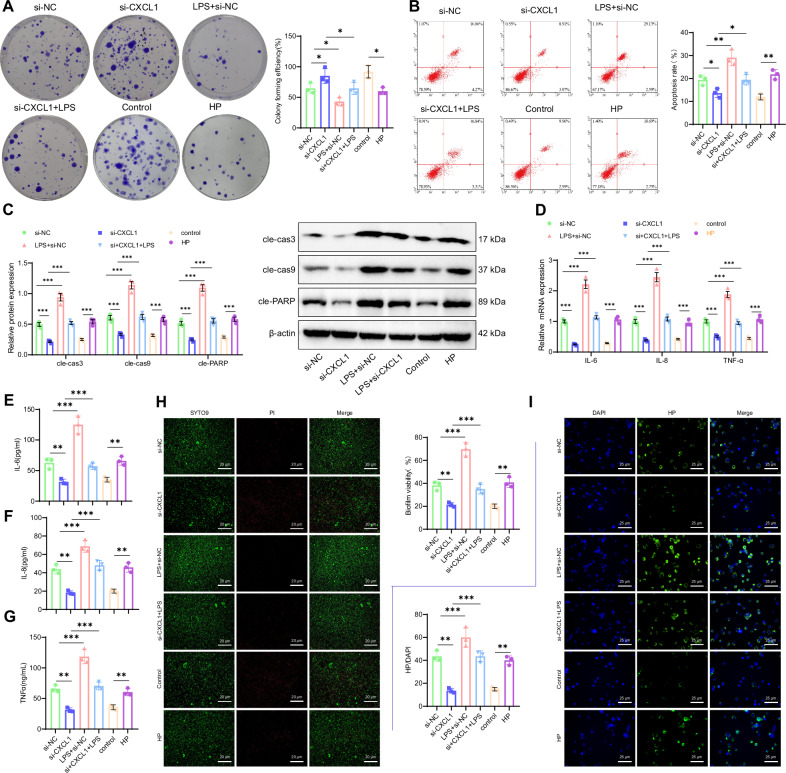
Impact of CXCL1/NF-κB signaling axis on HPI GES-1 cells. **A** Colony formation assay to detect the survival of HPI-treated GES-1 cells. **B** Flow cytometry analysis to detect apoptosis in HPI-treated GES-1 cells. **C** Western blot analysis of cleaved caspase-3, cleaved caspase-9, and cleaved PARP levels. **D** RT-qPCR analysis of IL-6, IL-8, and TNF-α mRNA levels in HPI-treated GES-1 cells. **E** ELISA analysis of IL-6 levels in HPI-treated GES-1 cells. **F** ELISA analysis of IL-8 levels in HPI-treated GES-1 cells. **G** ELISA analysis of TNF-α levels in HPI-treated GES-1 cells. **H** Immunofluorescence staining to detect biofilm formation in HPI-treated GES-1 cells, scale bar: 25 μm; **I** Immunofluorescence staining to detect HP adhesion in HPI-treated GES-1 cells, scale bar: 25 μm. Values are presented as the mean ± standard deviation, and all cell experiments were repeated three times. *** indicates *P* < 0.001.

Next, we explored the effect of the CXCL1/NF-κB signaling axis on the phenotype of HPI-treated GES-1 cells. First, colony formation assay results showed that, compared to the Control group, the colony-forming ability of the HP group was significantly reduced. Compared to the si-NC group, the colony-forming ability of the si-CXCL1 group was significantly increased, while the colony-forming ability of the LPS+si-NC group was significantly reduced. Compared to the si-CXCL1 group, the colony-forming ability of the si-CXCL1 + LPS group was significantly reduced (Fig. 5A). Next, flow cytometry results showed that, compared to the Control group, the apoptosis rate in the HP group was significantly increased. Compared to the si-NC group, the apoptosis rate in the si-CXCL1 group was significantly reduced, while the apoptosis rate in the LPS+si-NC group was significantly increased. Compared to the si-CXCL1 group, the apoptosis rate in the si-CXCL1 + LPS group was significantly increased (Fig. 5B). Meanwhile, WB results showed that, compared to the Control group, the levels of cleaved caspase-3, cleaved caspase-9, and cleaved PARP were elevated in the HP group. Compared to the si-NC group, the levels of cleaved caspase-3, cleaved caspase-9, and cleaved PARP were reduced in the si-CXCL1 group, while they were significantly increased in the LPS+si-NC group. Compared to the si-CXCL1 group, the levels of cleaved caspase-3, cleaved caspase-9, and cleaved PARP were elevated in the si-CXCL1 + LPS group (Fig. 5C). Next, RT-qPCR and ELISA results showed that, compared to the Control group, the mRNA expression levels and content of IL-6, IL-8, and TNF-α were significantly increased in the HP group. Compared to the si-NC group, the mRNA expression levels and content of IL-6, IL-8, and TNF-α were significantly reduced in the si-CXCL1 group, while they were significantly increased in the LPS+si-NC group. Compared to the si-CXCL1 group, the mRNA expression levels and content of IL-6, IL-8, and TNF-α were significantly increased in the si-CXCL1 + LPS group (Fig. 5D–G).

Finally, immunofluorescence staining results for biofilm formation showed that, compared to the Control group, the green fluorescence was enhanced in the HP group, indicating the formation of a mature biofilm. Compared to the si-NC group, the green fluorescence in the si-CXCL1 group was reduced, indicating biofilm disintegration, while the green fluorescence in the LPS+si-NC group was enhanced, indicating the formation of a mature biofilm. Compared to the si-CXCL1 + LPS group, biofilm formation was increased in the CXCL1 + LPS group (Fig. 5H). Immunofluorescence staining results for HP adhesion showed that, compared to the Control group, the green fluorescence in the HP group was enhanced, and HP adhesion was significantly increased. Compared to the si-NC group, the green fluorescence in the si-CXCL1 group was reduced, and HP adhesion was significantly decreased, while the green fluorescence in the LPS+si-NC group was enhanced, and HP adhesion was significantly increased. Compared to the si-CXCL1 group, HP adhesion was increased in the si-CXCL1 + LPS group (Fig. 5I).

In summary, our results indicate that CXCL1 activates the NF-κB signaling pathway, further exacerbating cell apoptosis, biofilm formation, HP adhesion, and the inflammatory response induced by HPI.

### METTL3 activates the CXCL1/NF-κB axis to induce inflammation in HPI-treated GES-1 Cells

**Fig. 6:**
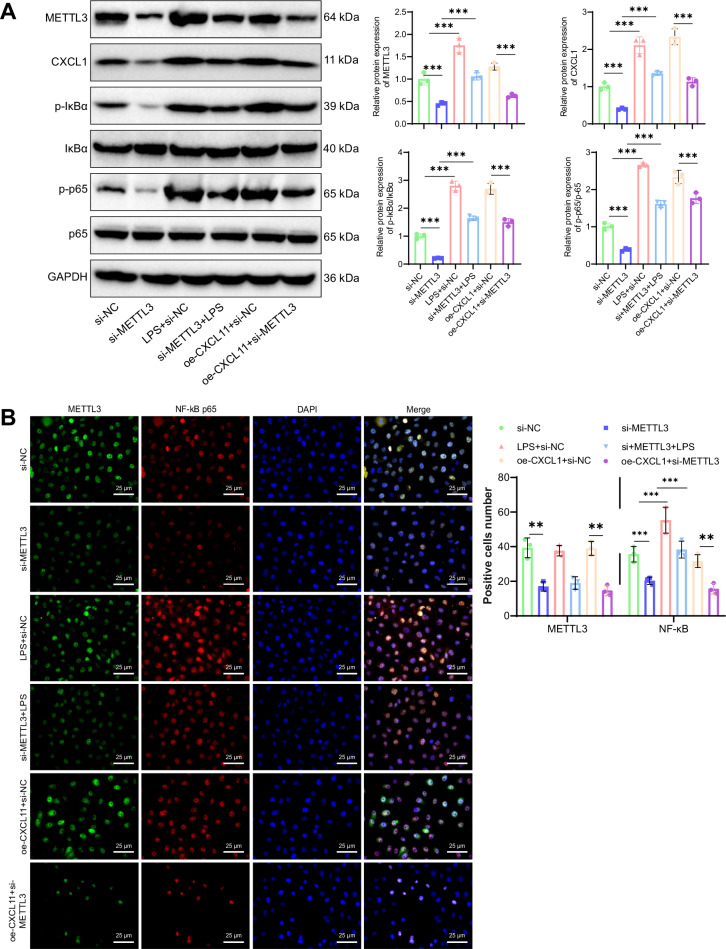
Validation of the relationship between METTL3 and the NF-κB signaling pathway. **A** Western blot analysis of the protein expression levels of p-IκBα, IκBα, p-p65, and p65 in HPI GES-1 cells; bar graphs represent the ratios of p-IκBα/IκBα and p-p65/p65 protein levels. **B** Immunofluorescence staining to detect the expression levels of METTL3 and NF-κB in HPI GES-1 cells, scale bar: 25 μm. Values are presented as mean ± standard deviation; all cell experiments were repeated three times, and *** indicates *P* < 0.001.

METTL3 Activation of the CXCL1/NF-κB Signaling Axis Promotes Inflammatory Response in HPI GES-1 Cells. Previous research has indicated that METTL3 promotes CXCL1 expression through RNA m6A modification, leading to the activation of the NF-κB signaling pathway. To further investigate whether METTL3 can activate the NF-κB signaling pathway by upregulating CXCL1 expression, the following experiments were conducted. Western blot results showed that compared to the si-NC group, the si-METTL3 group exhibited significant downregulation of METTL3 and CXCL1 protein levels, with a slight decrease in the p-IκBα/IκBα and p-p65/p65 ratios. In the LPS+si-NC group, there were no significant changes in METTL3 and CXCL1 protein levels, while the protein levels of p-IκBα/IκBα and p-p65/p65 ratios were significantly upregulated. Moreover, compared to the si-METTL3 group, both the si-METTL3 + LPS and si-METTL3+oe-CXCL11 groups showed a partial increase in the protein levels of p-IκBα/IκBα and p-p65/p65 ratios (Fig. 6A). Immunofluorescence staining results showed that, compared to the si-NC group, the p-p65 fluorescence intensity was reduced in the si-METTL3 group and increased in the LPS+si-NC group. Compared to the si-METTL3 group, the fluorescence intensity was increased in the si-METTL3 + LPS group and the si-METTL3+oe-CXCL11 group. These results suggest that si-METTL3 reduces the nuclear translocation of p65, thereby inhibiting the NF-κB signaling pathway, which corroborates the Western blot results (Fig. 6B).

**Fig. 7:**
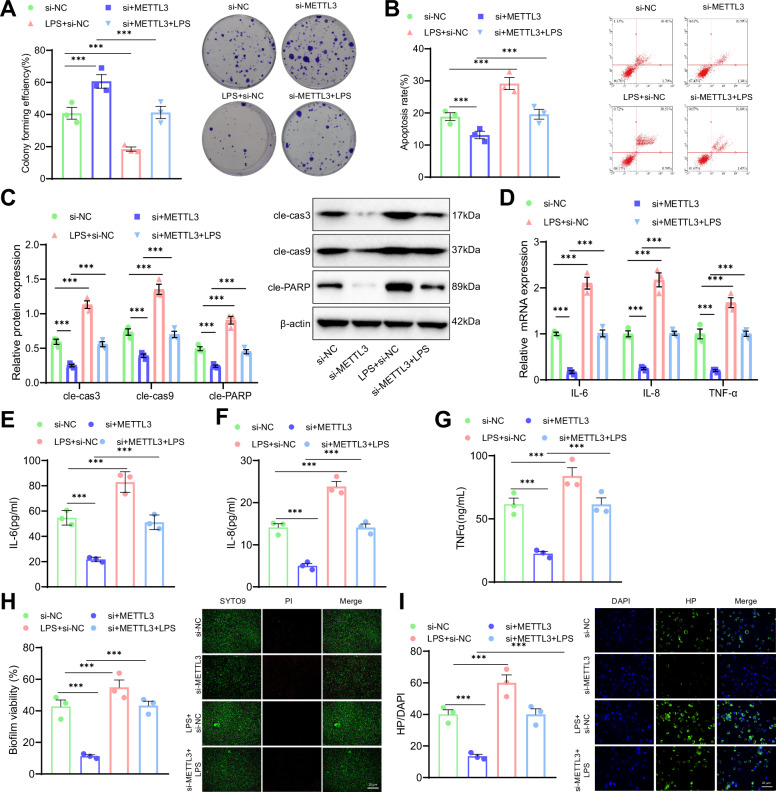
Impact of METTL3/NF-κB signaling axis on HPI GES-1 cells. **A** Colony formation assay to detect the survival of HPI-treated GES-1 cells. **B** Flow cytometry analysis to detect apoptosis in HPI-treated GES-1 cells. **C** Western blot analysis of cleaved caspase-3, cleaved caspase-9, and cleaved PARP levels. **D** RT-qPCR analysis of IL-6, IL-8, and TNF-α mRNA levels in HPI-treated GES-1 cells. **E** ELISA analysis of IL-6 levels in HPI-treated GES-1 cells. **F** ELISA analysis of IL-8 levels in HPI-treated GES-1 cells. **G** ELISA analysis of TNF-α levels in HPI-treated GES-1 cells. **H** Immunofluorescence staining to detect biofilm formation in HPI-treated GES-1 cells, scale bar:25 μm; **I** Immunofluorescence staining to detect HP adhesion in HPI-treated GES-1 cells, scale bar: 25 μm. Values are presented as the mean ± standard deviation, and all cell experiments were repeated three times. *** indicates *P* < 0.001.

Subsequently, the effects of the METTL3/CXCL1/NF-κB signaling axis on the phenotype of HPI GES-1 cells were investigated. Initially, the experimental results demonstrate a marked increase in the colony formation ability of the si-METTL3 group compared to the si-NC group. Conversely, the colony formation ability was decreased in the LPS+si-NC group. Furthermore, the colony formation ability in the si-METTL3 + LPS group was reduced compared to the si-METTL3 group (Fig. 7A). Furthermore, flow cytometry results demonstrated that the cell apoptosis rate decreased in the si-METTL3 group compared with the si-NC group; conversely, it increased in the LPS+si-NC group.

Additionally, the cell apoptosis rate increased in the si-METTL3 + LPS group relative to the si-METTL3 group (Fig. 7B). Meanwhile, Western blot results showed that, compared to the si-NC group, the levels of cleaved caspase-3, cleaved caspase-9, and cleaved PARP were reduced in the si-METTL3 group, while these levels were elevated in the LPS+si-NC group. Compared to the si-METTL3 group, the levels of cleaved caspase-3, cleaved caspase-9, and cleaved PARP were increased in the si-METTL3 + LPS group (Fig. 7C). Furthermore, RT-qPCR and ELISA results showed that, compared to the si-NC group, the mRNA expression levels and content of IL-6, IL-8, and TNF-α were significantly increased in the si-METTL3 group, and the mRNA expression levels and content of IL-6, IL-8, and TNF-α were also significantly increased in the LPS+si-NC group. Compared to the si-METTL3 group, the mRNA expression levels and content of IL-6, IL-8, and TNF-α were significantly increased in the si-METTL3 + LPS group (Fig. 7D–G).

Finally, immunofluorescence staining results for biofilm formation showed that, compared to the si-NC group, the green fluorescence was reduced, and biofilm formation was disrupted in the si-METTL3 group. The green fluorescence in the LPS+si-NC group was significantly increased, indicating the formation of a mature biofilm. Compared to the METTL3 + LPS group, biofilm formation increased in the si-METTL3 + LPS group (Fig. 7H). Immunofluorescence staining results for HP adhesion showed that, compared to the si-NC group, the green fluorescence in the si-METTL3 group was reduced, and HP adhesion was decreased. In contrast, the green fluorescence in the LPS+si-NC group was enhanced, and HP adhesion increased. Compared to the si-METTL3 group, HP adhesion was increased in the si-METTL3 + LPS group (Fig. 7I).

In summary, these results demonstrate that METTL3 activates the NF-κB signaling pathway by upregulating CXCL1 expression, thereby exacerbating HPI-induced apoptosis, biofilm formation, HP adhesion, and the inflammatory response in GES-1 cells.

### METTL3 activation of the CXCL1/NF-κB pathway exacerbates inflammatory responses in HPI-induced gastritis: in vivo evidence

**Fig. 8:**
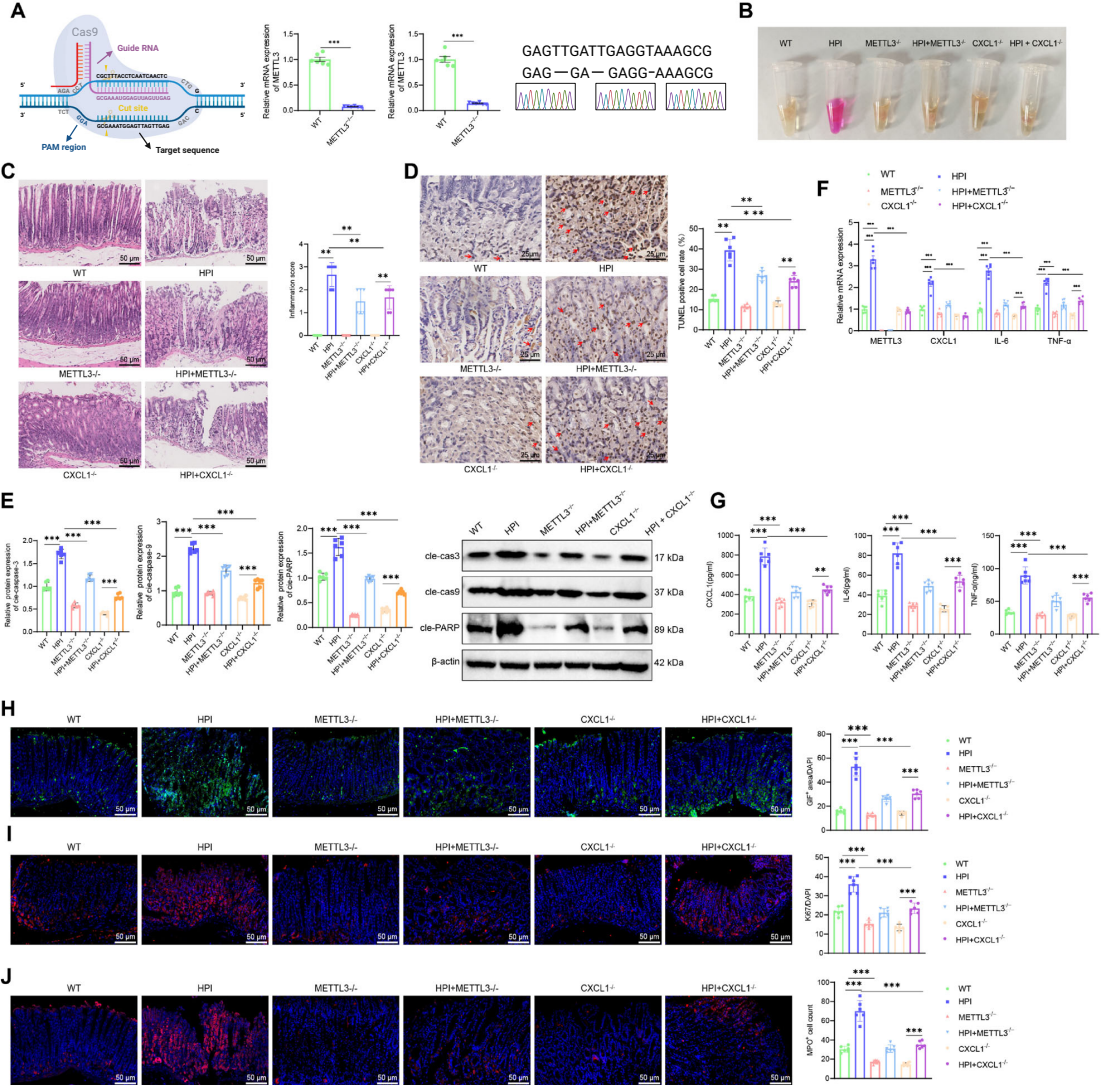
Effects of METTL3 knockout on HPI gastric inflammation in mice. **A** Construction of METTL3^−/−^ and CXCL1^−/−^ mice using CRISPR-Pro gene knockout technology, along with RT-qPCR and sequencing results. **B** Rapid urease test to detect gastric mucosal infection in mice. **C** H&E staining to observe the morphology and structure of gastric mucosal cells, scale bar: 50 μm. **D** TUNEL assay to detect apoptosis in gastric mucosal cells, scale bar: 25 μm. **E** Western blot analysis of cleaved caspase-3, cleaved caspase-9, and cleaved PARP levels. **F** RT-qPCR analysis of CXCL1, IL-6, IL-8, and TNF-α mRNA levels in the gastric mucosa of mice. **G** ELISA analysis of CXCL1, IL-6, and TNF-α levels in the gastric mucosa of mice. **H** Immunofluorescence detection of GIF expression in the gastric mucosa, scale bar: 25 μm. **I** Immunofluorescence detection of Ki67 expression in the gastric mucosa, scale bar: 25 μm. **J** Immunofluorescence detection of MPO expression in the gastric mucosa, scale bar: 25 μm. Each group contains 6 mice. Values are presented as the mean ± standard deviation, * indicates *P* < 0.05, ** indicates *P* < 0.01, *** indicates *P* < 0.001.

Finally, we established a mouse gastritis model using HP bacterial solution gavage to investigate the regulatory role of the METTL3/CXCL1/NF-κB signaling axis in vivo. First, METTL3^−/−^ and CXCL1^−/−^ mice were generated using CRISPR-Pro gene knockout technology. RT-qPCR and sequencing results confirmed successful knockout of METTL3 and CXCL1 (Fig. 8A). To evaluate whether the model was successfully established, we conducted a rapid urease test and H&E staining. The rapid urease test results showed that the solution in the reagent tubes containing gastric tissue from the WT, METTL3^−/−^, and CXCL1^−/−^ groups remained yellow without change, while the solution from the HPI group turned from yellow to purple-red. In the HPI + METTL3^−/−^ and HPI + CXCL1^−/−^ groups, the reagent solution remained yellow (Fig. 8B). H&E staining results showed that the gastric mucosa in the WT, CXCL1^−/−^, and METTL3^−/−^ groups appeared smooth, with orderly glandular arrangement and no swelling or hemorrhage. In the HPI group, the gastric mucosa appeared less smooth, with some epithelial necrosis and detachment, significant congestion and swelling, disordered glandular arrangement, and scattered hemorrhage, with infiltration of lymphocytes, plasma cells, and a few neutrophils. In the HPI + METTL3^−/−^ and HPI + CXCL1^−/−^ groups, mucosal epithelial damage was significantly reduced, and the glandular arrangement remained orderly (Fig. 8C). These results indicated that the HPI gastritis mouse model was successfully established, and knockout of METTL3 and CXCL1 alleviated HPI-induced gastritis symptoms.

Next, we investigated the effect of METTL3 knockout on the phenotype of HPI-induced gastritis in mice. First, WB and TUNEL assay results showed low background staining in the gastric mucosa of WT mice, with clear nuclear staining and few or no brown crystals. In contrast, the HPI group showed weak nuclear staining and many brown crystals. The METTL3^−/−^, CXCL1^−/−^, HPI + METTL3^−/−^, and HPI + CXCL1^−/−^ groups had fewer brown crystals, suggesting that METTL3 and CXCL1 knockdown reduced apoptosis in the gastric mucosa (Fig. 8D). Meanwhile, WB results showed that, compared to the WT group, the levels of cleaved caspase-3, cleaved caspase-9, and cleaved PARP were upregulated in the gastric mucosa of HPI mice. These levels were slightly reduced in the METTL3^−/−^ and CXCL1^−/−^ groups. Compared to the HPI group, the levels of cleaved caspase-3, cleaved caspase-9, and cleaved PARP were significantly reduced in the HPI + METTL3^−/−^ and HPI + CXCL1^−/−^ groups (Fig. 8E). RT-qPCR results showed that, compared to the WT group, METTL3, CXCL1, IL-6, and TNF-α mRNA expression levels were upregulated in the gastric mucosa of HPI mice. METTL3 was not expressed in the METTL3^−/−^ group, and CXCL1, IL-6, and TNF-α mRNA levels were slightly reduced in both the METTL3^−/−^ and CXCL1^−/−^ groups. Compared to the HPI group, METTL3 was not expressed, and CXCL1, IL-6, and TNF-α mRNA levels were significantly reduced in the HPI + METTL3^−/−^ and HPI + CXCL1^−/−^ groups (Fig. 8F). ELISA results showed that, compared to the WT group, serum levels of CXCL1, IL-6, and TNF-α were significantly elevated in HPI mice. In the METTL3^−/−^ and CXCL1^−/−^ groups, IL-6 and TNF-α levels were slightly reduced. Compared to the HPI group, serum IL-6 and TNF-α levels were significantly reduced in the HPI + METTL3^−/−^ and HPI + CXCL1^−/−^ groups (Fig. 8G).

GIF secretion reflects gastric acid secretion, bacterial infection, and gastric mucosal damage [55]. Ki67 is a nuclear antigen, and Ki67+ immunostaining shows the expression and distribution of Ki67 protein in cells, indicating proliferative activity [56]. MPO is an enzyme found in neutrophils that is mainly involved in immune response and inflammation [57]. Immunofluorescence staining results showed that, compared to the WT group, the areas positive for GIF + , Ki67 + , and MPO+ were significantly increased in the gastric mucosa of HPI mice, while these areas were slightly reduced in the METTL3^−/−^ and CXCL1^−/−^ groups. Compared to the HPI group, the GIF + , Ki67 + , and MPO+ areas were significantly reduced in the gastric mucosa of the HPI + METTL3^−/−^ and HPI + CXCL1^−/−^ groups (Fig. 8H–J). These findings suggest that METTL3 and CXCL1 knockout reduced gastric mucosal cell proliferation and neutrophil infiltration. Taken together, METTL3 knockdown alleviated apoptosis and inflammation in the gastric mucosa of HPI gastritis mice.

**Fig. 9:**
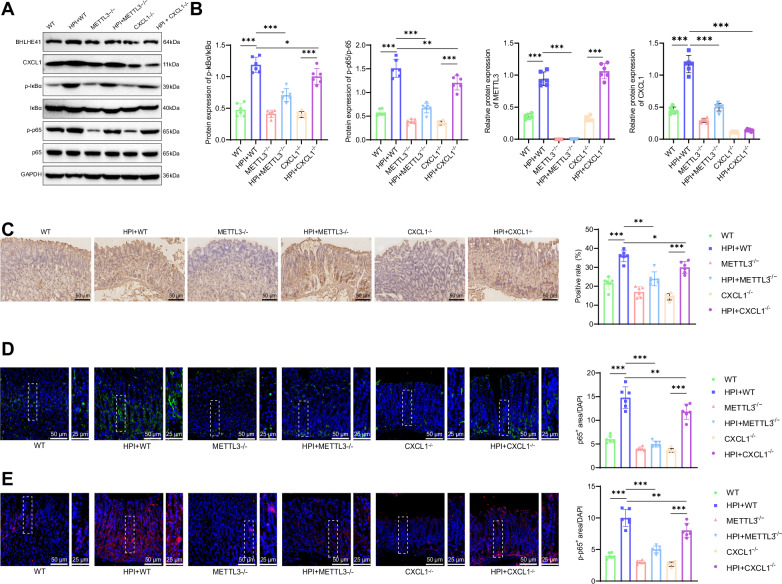
Effects of METTL3 knockout on the CXCL1/NF-κB signaling axis in HPI gastric inflammation mice. **A** Western blot analysis of the protein levels of METTL3, CXCL1, p-IκBα, IκBα, p-p65, and p65 in gastric mucosa of mice. **B** METTL3, CXCL1 and ratios of p-IκBα/IκBα, p-p65/p65 protein levels. **C** Immunohistochemical staining to detect the expression of CXCL1 in gastric mucosa of mice, scale bar: 50 μm. **D** Immunofluorescence staining to assess the expression levels of p65 in gastric mucosa of mice, with the right images showing enlarged views of the left images, scale bar: 50 /25 μm. **E** Immunofluorescence staining to assess the expression levels of p-p65 in gastric mucosa of mice, with the right images showing enlarged views of the left images, scale bar: 50 /25 μm. Each group consisted of 6 mice, values are presented as mean ± standard deviation, * indicates *P* < 0.05, ** indicates *P* < 0.01, *** indicates *P* < 0.001.

Finally, we investigated whether HP exacerbates the inflammatory response in gastritis mice through the METTL3/CXCL1/NF-κB signaling axis. First, Western blot results showed that, compared with the WT group, the expression of METTL3, CXCL1, p-IκBα/IκBα, and p-p65/p65 protein ratios were significantly upregulated in the gastric mucosal tissues of the HPI + WT group. In the METTL3^−/−^ group, the expression of CXCL1, p-IκBα/IκBα, and p-p65/p65 protein ratios in the gastric mucosa were slightly downregulated, while in the CXCL1^−/−^ group, the expression of p-IκBα/IκBα and p-p65/p65 protein ratios was slightly downregulated, with METTL3 protein levels unaffected. Compared to the HPI + WT group, in the HPI + METTL3^−/−^ group, the expression of CXCL1, p-IκBα/IκBα, and p-p65/p65 protein ratios in the gastric mucosa was significantly downregulated, and in the HPI + CXCL1^−/−^ group, the expression of p-IκBα/IκBα and p-p65/p65 protein ratios in the gastric mucosa was significantly downregulated, with METTL3 protein levels unaffected (Fig. 9A, B).

Next, immunohistochemistry results showed that compared to the WT group, the HPI + WT group exhibited a large amount of brown-yellow staining, with high expression of CXCL1 protein in the gastric mucosa. In the METTL3^−/−^ and CXCL1^−/−^ groups, there was no significant brown-yellow staining in the gastric mucosa. Compared to the HPI + WT group, the HPI + METTL3^−/−^ and HPI + CXCL1^−/−^ groups exhibited reduced brown-yellow staining, and the expression of CXCL1 protein was downregulated (Fig. 9C).

Finally, immunofluorescence results showed that the HPI + WT group exhibited an increased trend of p65 nuclear translocation and upregulated p-p65 protein levels in the gastric mucosa. In the METTL3^−/−^ and CXCL1^−/−^ groups, there was a slight decrease in p65 nuclear translocation and p-p65 protein levels. Compared to the HPI + WT group, the HPI + METTL3^−/−^ and HPI + CXCL1^−/−^ groups showed a significant decrease in the trend of p65 nuclear translocation and a downregulation of p-p65 protein levels in the gastric mucosa (Fig. 9D, E).

In conclusion, METTL3 upregulates CXCL1 expression through m6A modification, activating the NF-κB signaling pathway and exacerbating the inflammatory response in HPI-induced gastritis in mice.

## Discussion

HP is a contributor to Gastritis and gastric ulcers, and persistent infection is also linked to the development of gastric cancer [2–4, 6]. The inflammatory response plays a central role in the pathogenesis of HP infection [58]. Previous studies have revealed that the NF-κB signaling pathway is strongly linked to inflammatory responses. However, the specific regulatory mechanisms underlying this association have not yet been fully elucidated [19–21]. This study emphasizes the crucial role of METTL3 in regulating m6A modification on CXCL1 throughout this process.

A positive correlation was discovered between METTL3 and CXCL1 in the gastric mucosa of patients infected with HPI in this study. This finding is consistent with previous studies regarding the regulatory role of METTL3 in other inflammatory reactions and tumors [59, 60]. However, it sheds new light on the specific mechanism of METTL3 in HP-infected Gastritis, which has not been reported before [13, 14, 61]. This discovery offers a novel functional perspective on METTL3 and provides valuable insights for further exploration of therapeutic strategies for HP infection. This study provides evidence that METTL3 promotes CXCL1 expression through m6A modification in vitro cell experiments, leading to the activation of the NF-κB signaling pathway. This finding offers novel insights into the involvement of NF-κB in HP infection, thereby enhancing our comprehension of this integral signaling pathway in inflammation. Simultaneously, it also highlights the significance of m6A modification in this process. After HP treatment, CXCL1 expression in GES-1 cells was significantly increased (Fig. 2A, B). CXCL1 is a chemokine that is typically associated with inflammatory responses and immune reactions. Previous studies have shown that elevated CXCL1 expression can exacerbate bacterial proliferation [62, 63], thereby increasing HP bacterial load. This suggests that HP may increase bacterial load by regulating METTL3 to elevate CXCL1 expression, which could be part of HP’s self-survival mechanism.

Early studies have reported that the NF-κB signaling pathway is crucial in HP-induced gastritis [64]. However, there is still a need for further in-depth research to investigate the specific regulation mechanisms, particularly concerning post-transcriptional modifications. This study confirms the roles of METTL3 and CXCL1 in HP-induced Gastritis while also elucidating their connection to the NF-κB signaling pathway. This study conducted a comprehensive set of rigorous experiments, from bioinformatics analysis to in vitro cell experiments and, ultimately, in vivo animal models. These experiments provide robust evidence to support the research findings. Specifically, the study’s findings were further verified through in vivo experiments that suppressed METTL3 and observed its impact on gastric mucosal cells in HPI mice. Based on the findings of this study, METTL3 could potentially serve as a novel therapeutic target for the management of HP-induced Gastritis. By disrupting the activity of METTL3 or its modifications on CXCL1, patients may be offered novel therapeutic strategies. Nevertheless, this study is currently in its preliminary stages, and additional experimental validation and comprehensive research will be necessary to facilitate the application of this discovery in a clinical environment.

NF-κB is generally considered to negatively regulate apoptosis by promoting the transcription of anti-apoptotic genes. However, in specific cell types, under environmental stress, or within certain interaction networks, it may participate in pro-apoptotic processes under specific conditions. For example, during inflammation, NF-κB activation may lead to the release of inflammation-related cytokines, thereby exacerbating cell damage and apoptosis [65]. Moreover, there are complex interactions between the NF-κB pathway and other signaling pathways. Under particular physiological or pathological conditions, reducing the activation of one pathway (such as the NF-κB signaling axis) can shift the intracellular signaling balance, potentially leading to reduced levels of apoptosis [66, 67].

Based on the above findings, we could preliminarily deduce the following conclusions: METTTL3 enhances the activation of the NF-κB signaling pathway, leading to apoptosis and inflammatory response in HPI gastritis through RNA m6A modification of CXCL1 (Fig. S4).

This study uncovers the role of METTL3 in regulating CXCL1 expression through m6A modification, thereby activating the NF-κB signaling pathway in the pathogenesis of HP-induced gastritis. It provides a novel understanding of the molecular mechanisms involved in HP-induced Gastritis. The correlation between METTL3 and CXCL1 suggests a potential therapeutic target for HP-induced gastritis [22]. Regulating this pathway could provide more precise and effective treatment options for patients with Gastritis. The signaling pathway involving METTL3-CXCL1-NF-κB is not limited to HP-infected gastritis alone. This pathway may also influence other inflammatory diseases, offering novel treatment strategies for these conditions.

The preliminary findings of this study, which utilized the GEO database and other bioinformatics tools, indicate that despite their overall usefulness, these tools may still introduce biases or lead to misinterpretations. The data obtained in GES-1 cells may differ from the in vivo environment. Therefore, it is necessary to further validate these in vitro experimental results using animal models and human samples. Although this study primarily investigates the METTL3-CXCL1-NF-κB signaling pathway, there could be additional unidentified signaling pathways that regulate the development and advancement of Gastritis. In the future, further validation and a more comprehensive understanding of the role of METTL3 in HP-induced Gastritis could be attained by increasing the utilization of databases and tools. Building upon animal models, this study provides additional confirmation of the precise function of the METTL3-CXCL1-NF-κB signaling pathway in HP-induced gastritis and also investigates its potential implications for other diseases. Suppose the results of animal model studies are positive. In that case, clinical trials may be potentially undertaken to assess the advantages of treatment strategies that aim at this pathway for patients with HP-induced gastritis.

Overall, this study yields valuable data for the scientific research community and introduces a fresh treatment perspective to clinical doctors. Despite its inherent limitations, the prospects appear promising.

## Supplementary information

original data-qPCR dat

Supplementary material

## Data Availability

The data that supports the findings of this study are available on request from the corresponding author upon reasonable request.
