# Supplementary material for: Unraveling the Role of METTL3 in Helicobacter pylori-induced gastritis via m6A-CXCL1/NF-κB modulation
Source: Cell Death Dis. 2025 Aug 18;16(1):625. doi: 10.1038/s41419-025-07841-4 (PMC12361391; doi:10.1038/s41419-025-07841-4)
Supplement: Supplementary file 2 — Supplementary material [file 41419_2025_7841_MOESM2_ESM.pdf]

Table S1. Sample Information of GEO Database Chip Dataset

Dataset	Platform	Sample
GSE5081	GPL570	8 samples of healthy volunteers and 8 samples of patients with H. pylori infection-related gastritis gastric tissue
GSE60427	GPL17077	8 samples of healthy volunteers and 16 samples of patients with H. pylori infection-related gastritis gastric tissue
GSE60662	GPL13497	4 samples of healthy volunteers and 8 samples of patients with H. pylori infection-related gastritis gastric tissue

Table S2. si-RNA primer sequences

Plasmids	Sequences (5'-3')
si-NC	GATCGTACTCACATCCACACT
si-METTL3-1	ATGTTGATCTGGAGATAGAGAGC
si-METTL3-2	AGGAACAATCCATTGTTGAAAAA
si-METTL3-3	GGGGTATGAACGGGTAGATGAAA
si-CXCL1-1	TAGTTCAATCTGGATTCATATTT
si-CXCL1-2	GGCCAATGAGATCATTGTGAAGG
si-CXCL1-3	CAGTGTTTCTGGCTTAGAACAAA
si-BHLHE4	CCGCACAGATTAATAGAAATT

Table S3. Grouping information of HPI GES-1 cells

Serial number	Group	Processing method
1	si-NC+oe-NC	Transfecting interfering and overexpressing empty plasmids into HPI GES-1 cells
2	si-METTL3+oe-NC	Transfecting HPI GES-1 cells with METTL3 interference and overexpressing empty plasmids
3	oe-CXCL1+si-NC	Transfecting HPI GES-1 cells with overexpressed CXCL1 and interference empty plasmids
4	si-METTL3+oe-CXCL1	Transfecting HPI GES-1 cells with METTL3 interference and CXCL1 overexpression plasmids
5	si-NC	Transfecting interfering empty plasmids into HPI GES-1 cells
6	si-CXCL1	Transfect the interference CXCL1 plasmid into HPI GES-1 cells
7	LPS+si-NC	Treat HPI GES-1 cells with LPS and transfect them with interference empty plasmids
8	si-CXCL1+LPS	Transfect interference CXCL1 into HPI GES-1 cells and treat with LPS
9	si-METTL3	Transfect the interference METTL3 plasmid into HPI GES-1 cells
10	si-METTL3+LPS	Transfecting HPI GES-1 cells with interfered METTL3 and treating with LPS

Table S4. The RT-qPCR primer sequence for CXCL1 m6A peak

m6A peak	primer sequence (5'-3')
CXCL1 m6A peak1	Forward: CCGAAGTCATAGCCACACTCA
	Reverse: TCAGGAACAGCCACCAGTGA
CXCL1 m6A peak2	Forward: GCCTCAATCCTGCATCCCC
	Reverse: CTGTTCCTATAAGGGCAGGGC
CXCL1 m6A peak3	Forward: CAATCCTGCATCCCCCATAGT
	Reverse: CAGGAACAGCCACCAGTGA

Table S5. RT-qPCR primer sequence

Gene	primer sequence (5’-3’)
GAPDH (Human)	Forward: GACAGTCAGCCGCATCTTCT
	Reverse: GCGCCCAATACGACCAAATC
METTL3 (Human)	Forward: ATCCCCAAGGCTTCAACCAG
	Reverse: GCGAGTGCCAGGAGATAGTC
CXCL1 (Human)	Forward: TGGCTTAGAACAAAGGGGCTT
	Reverse: GGTAGCCCTTGTTTCCCCC
IL-6 (Human)	Forward: CCACCGGGAACGAAAGAGAA
	Reverse: TCTTGTTACATGTTTGTGGAGAAGG
IL-8 (Human)	Forward: CACTGCGCCAACACAGAAA
	Reverse: TTCTCAGCCCTCTTCAAAAACTTC
TNF-α (Human)	Forward: AAAACAACCCTCAGACGCCA
	Reverse: TCCTTTCCAGGGGAGAGAGG
GAPDH (Mouse)	Forward: AAGAGGGATGCTGCCCTTAC
	Reverse: TACGGCCAAATCCGTTCACA
METTL3 (Mouse)	Forward: TGTTTCCATCCGTCTTGCCA
	Reverse: CATCTTGGAGGAGACCTCGC
CXCL1 (Mouse)	Forward: ACTCAAGAATGGTCGCGAGG
	Reverse: GTGCCATCAGAGCAGTCTGT
IL-6 (Mouse)	Forward: CCCCAATTTCCAATGCTCTCC
	Reverse: CGCACTAGGTTTGCCGAGTA
TNF-α (Mouse)	Forward: ATGGCCTCCCTCTCATCAGT
	Reverse: TTTGCTACGACGTGGGCTAC

Table S6. Gastric tissue scoring criteria

ANTRUM \ CORPUS	No Inflammation (score0)	Mild Inflammation (score1)	Moderate Inflammation (score2)	Severe Inflammation (score3)
No Inflammation (score0)	GRADE 0	GRADE II	GRADE II	GRADE II
Mild Inflammation(score1)	GRADE I	GRADE II	GRADE II	GRADE III
Moderate Inflammation (score2)	GRADE II	GRADE II	GRADE III	GRADE IV
Severe Inflammation (score3)	GRADE II	GRADE III	GRADE IV	GRADE IV

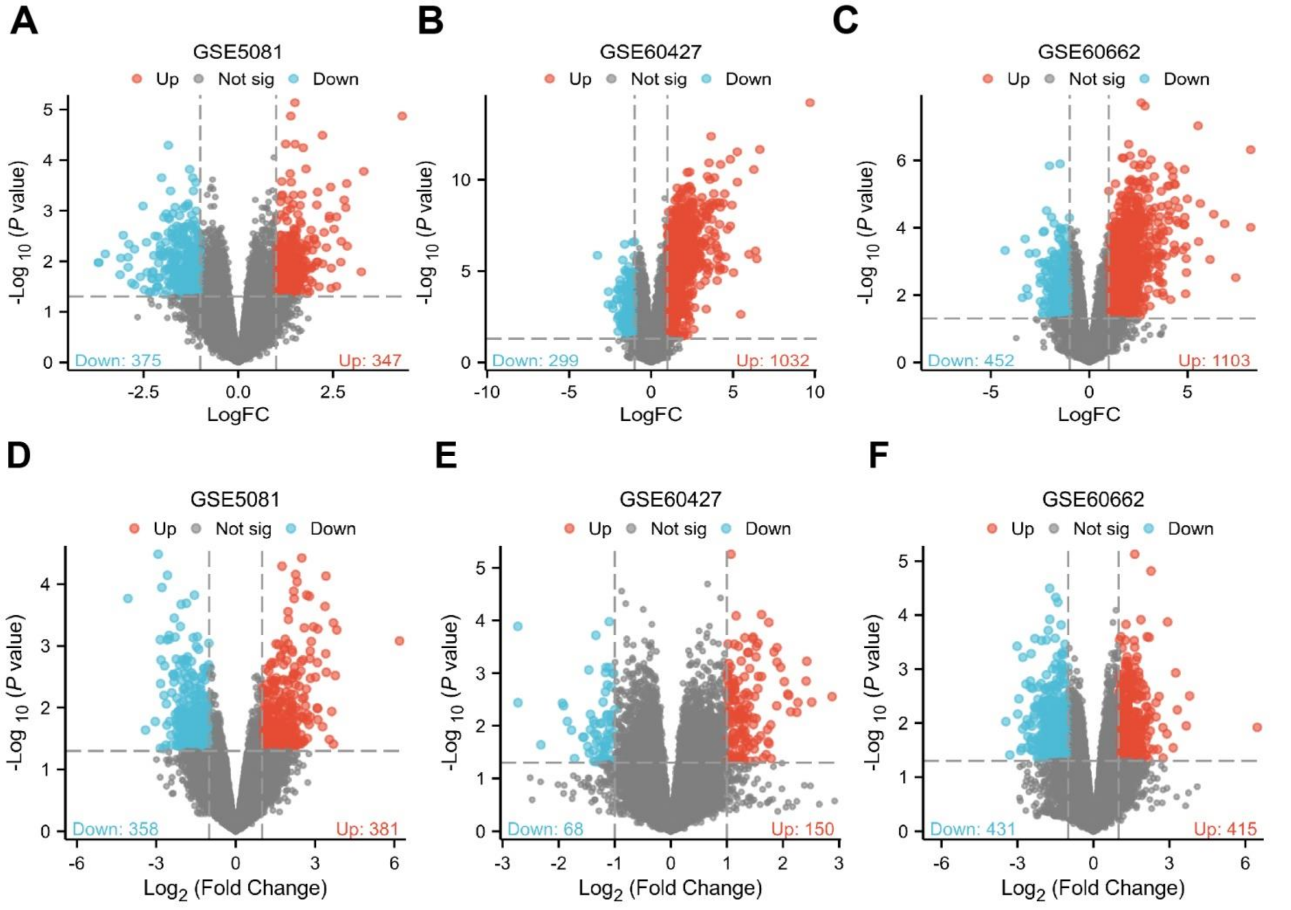

Fig. S1. Volcano plots showing differential gene expression in three HPI gastritis GEO datasets

Note: (A-C) Volcano plots showing differential gene expression between the Control group and Gastritis group in three HPI gastritis GEO datasets. (A) GSE5081 dataset, Control group: n=8; Gastritis group: n=8; (B) GSE60427 dataset, Control group: n=8; Gastritis group: n=16; (C) GSE60662 dataset, Control group: n=4; Gastritis group: n=8. Red dots represent genes significantly upregulated in the Gastritis group compared to the Control group, green dots represent genes significantly downregulated in the Gastritis group compared to the Control group, and black dots represent genes with no significant difference between the two groups. (D-F) Volcano plots showing differential gene expression between the CXCL1-Low group and CXCL1-High group in three HPI gastritis GEO datasets. (D) GSE5081 dataset, n=4; (E) GSE60427 dataset, n=8; (F) GSE60662 dataset, n=4. Red dots represent genes significantly upregulated in the CXCL1-High group compared to the CXCL1-Low group, green dots represent genes significantly downregulated in the CXCL1-High group compared to the CXCL1-Low group, and black dots represent genes with no

significant difference in expression between the CXCL1-Low and CXCL1-High groups.

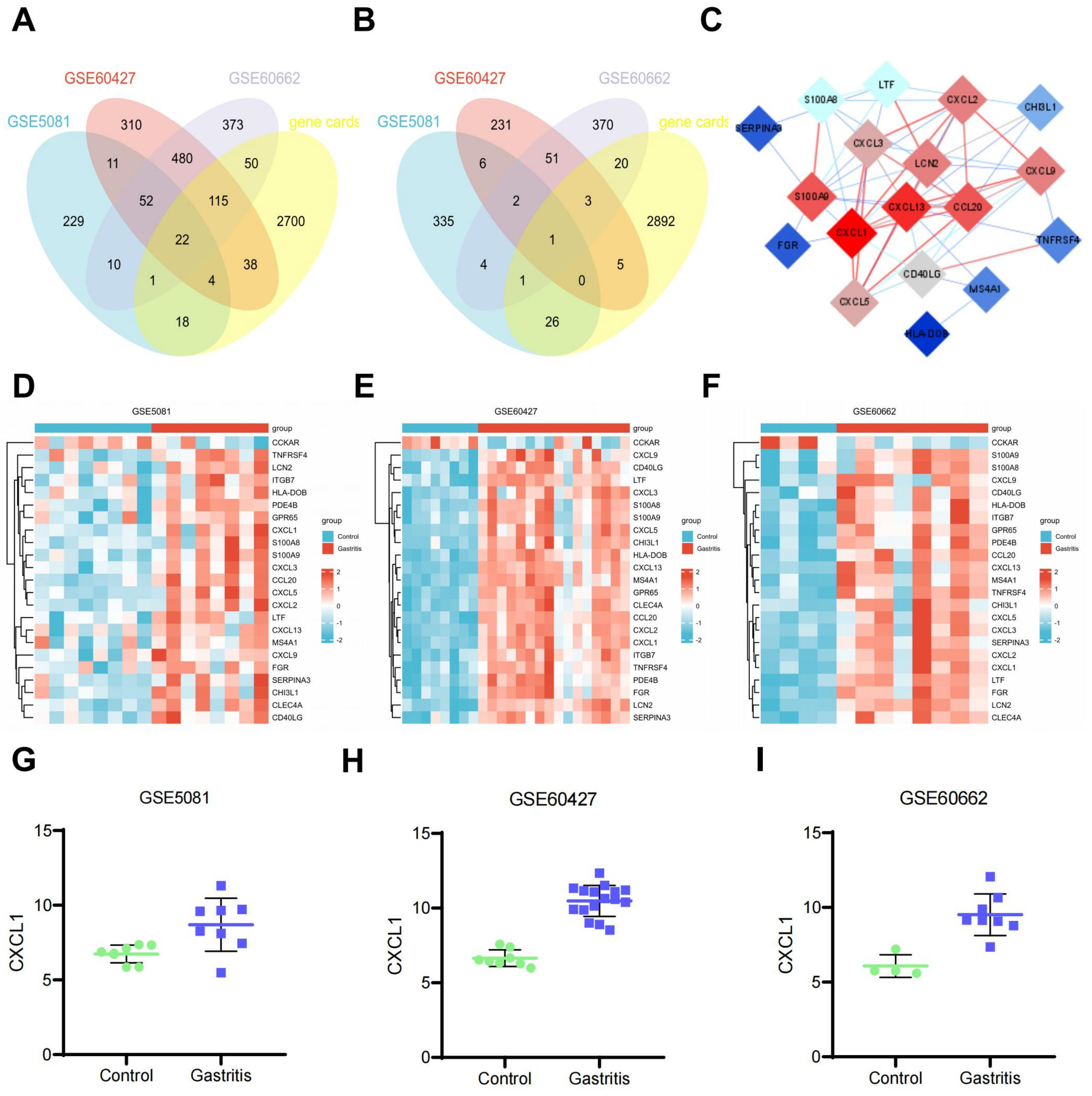

Fig. S2 Differential expression genes associated with HIP gastritis were identified from gastric tissue RNA sequencing data.

Note: (A) Venn diagram showing the intersection of upregulated genes in the Gastritis group across three HPI gastritis datasets with the HP-related gene set from GeneCards; (B) Venn diagram showing the intersection of downregulated genes in the Gastritis group across three HPI gastritis datasets with the HP-related gene set from GeneCards; (C) Construction of the PPI network for the 23 intersecting differentially expressed genes; (D) Expression profile of CXCL1 in the GSE5081 dataset; (E) Expression profile of CXCL1 in the GSE60427 dataset; (F) Expression profile of

CXCL1 in the GSE60662 dataset.

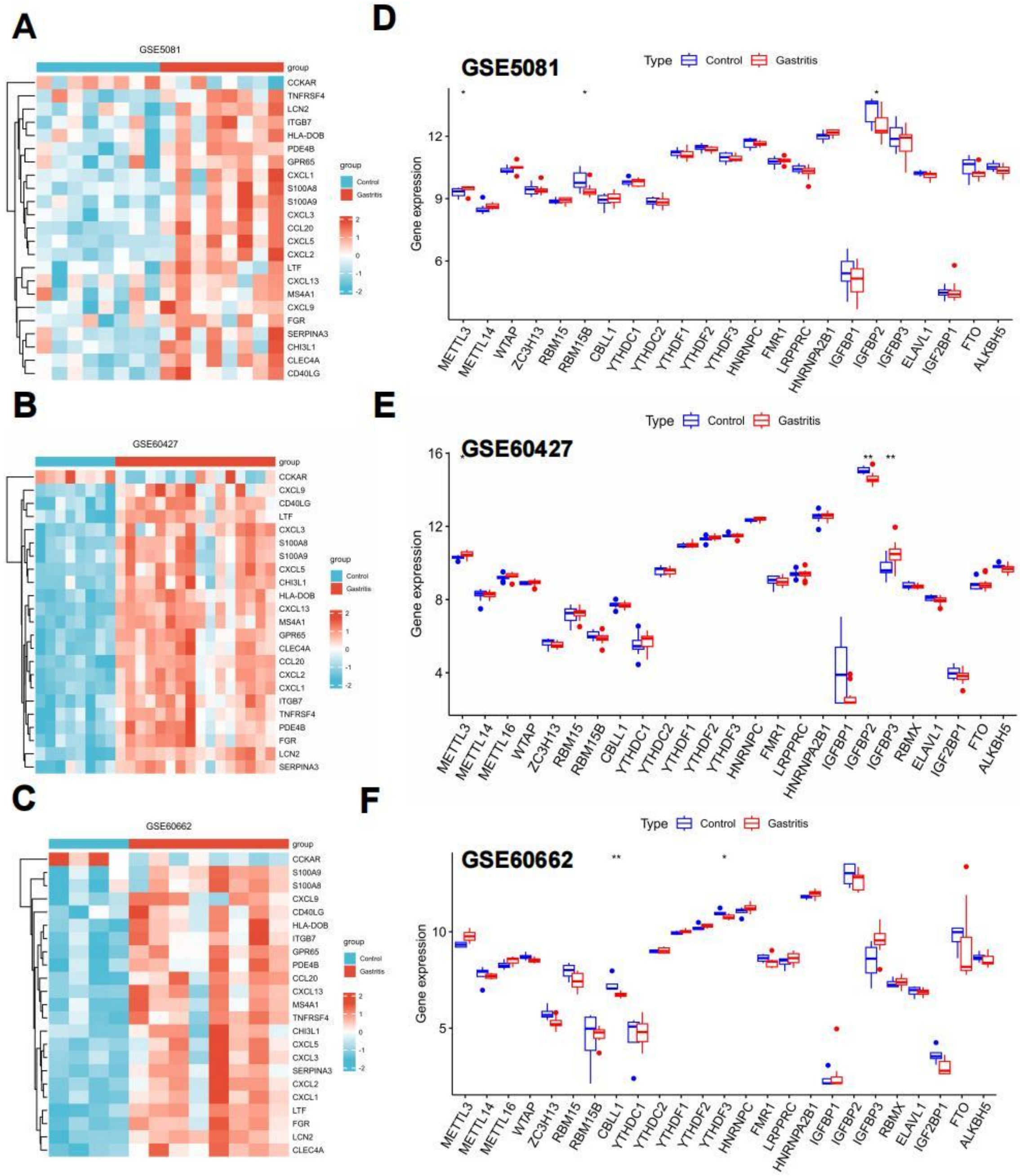

Fig. S3. Heatmap of GEO Data for HPI Gastritis.

Note: (A) Heatmap of the expression of the 23 intersecting differentially expressed genes in the GSE5081 dataset (Control group: n=8; Gastritis group: n=8); (B) Heatmap of the expression of the 23 intersecting genes in the GSE60427 dataset (Control group: n=8; Gastritis group: n=16); (C) Heatmap of the expression of the 23 intersecting genes in the GSE60662 dataset (Control group: n=4; Gastritis group: n=8); (D) Bar chart of the expression of m6A modification-related genes in the GSE5081 dataset (Control group: n=8; Gastritis group: n=8); (E) Bar chart of the expression of m6A modification-related genes in the GSE60427 dataset (Control group: n=8; Gastritis group:

n=16); (F) Bar chart of the expression of m6A modification-related genes in the GSE60662 dataset (Control group: n=4; Gastritis group: n=8).

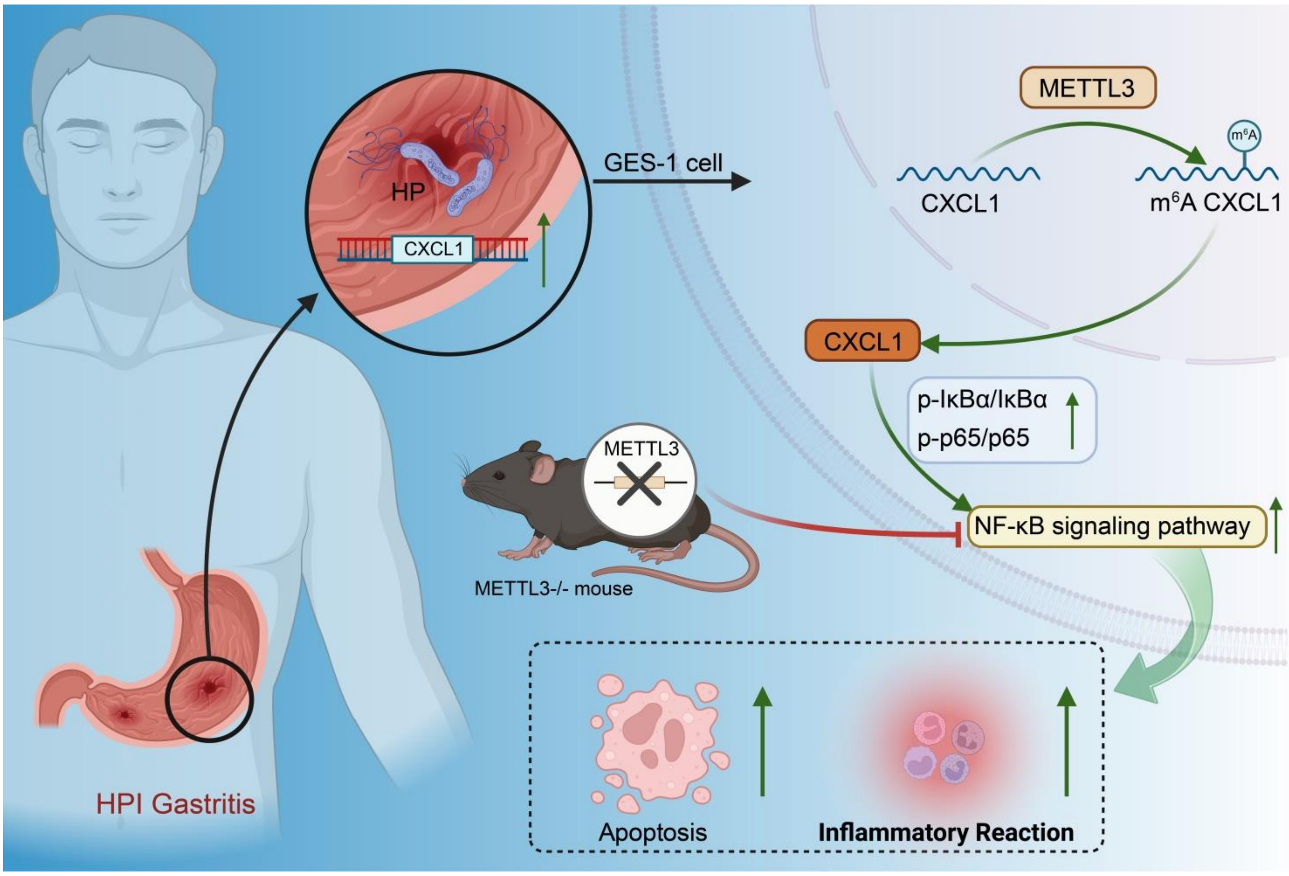

Fig. S4 Molecular mechanism diagram depicting the impact of METTL3 regulation on CXCL1 and NF-κB signaling in HPI gastric inflammation.

Full and uncropped western blots

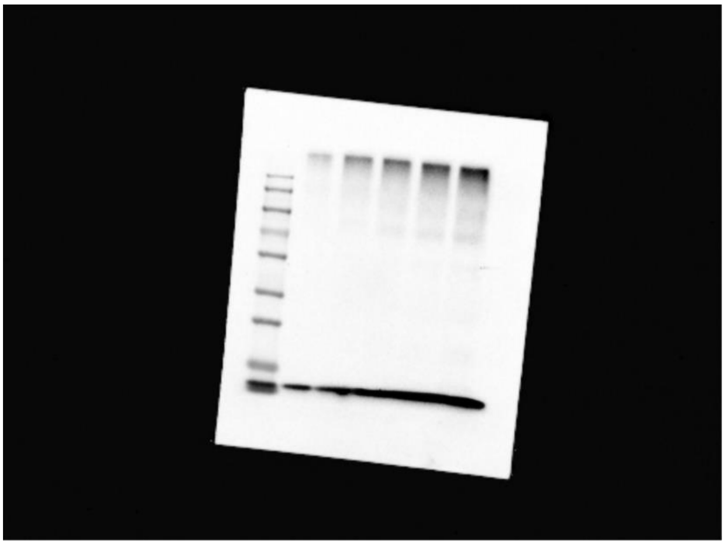

Full and uncropped western blots for Figure 2A-1

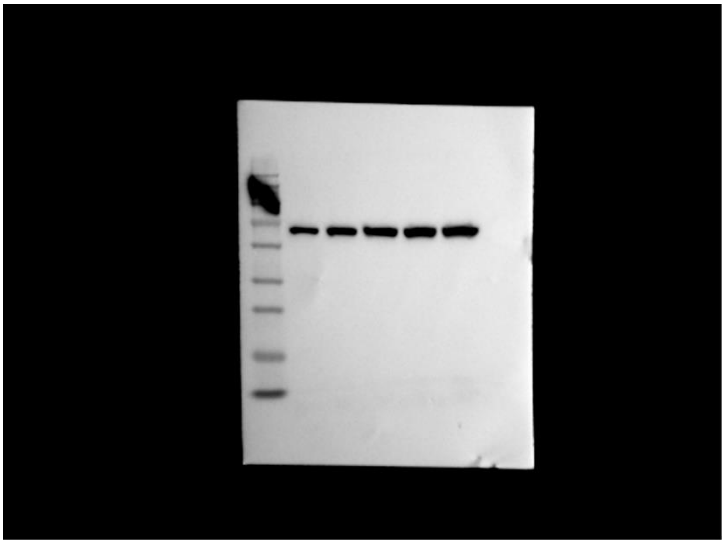

Full and uncropped western blots for Figure 2A-2

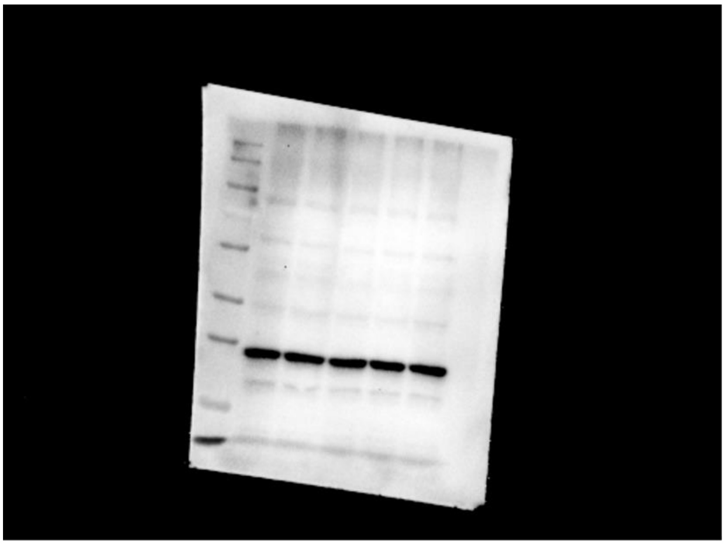

Full and uncropped western blots for Figure 2A-3

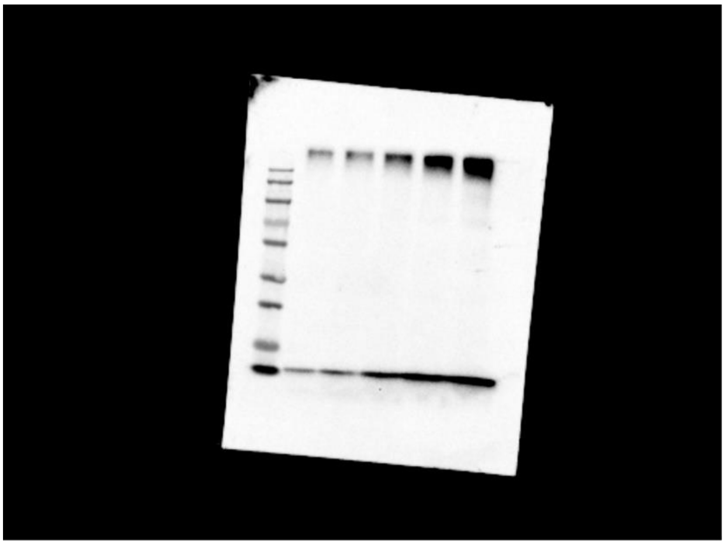
Full and uncropped western blots for Figure 2A-4
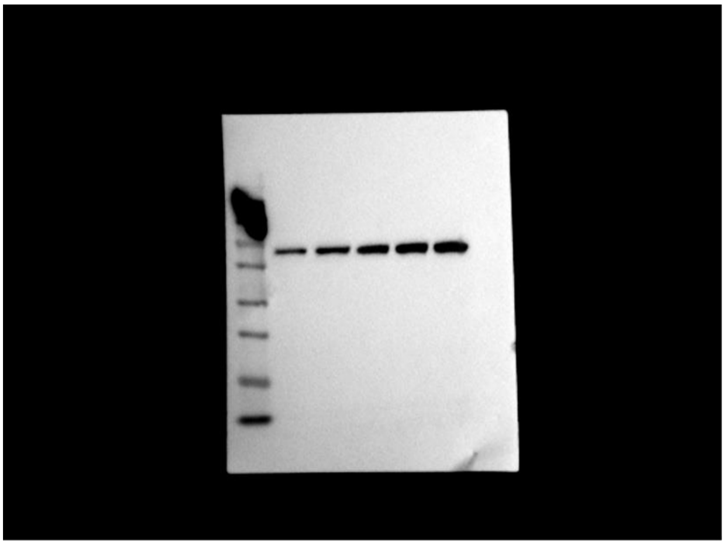
Full and uncropped western blots for Figure 2A-5
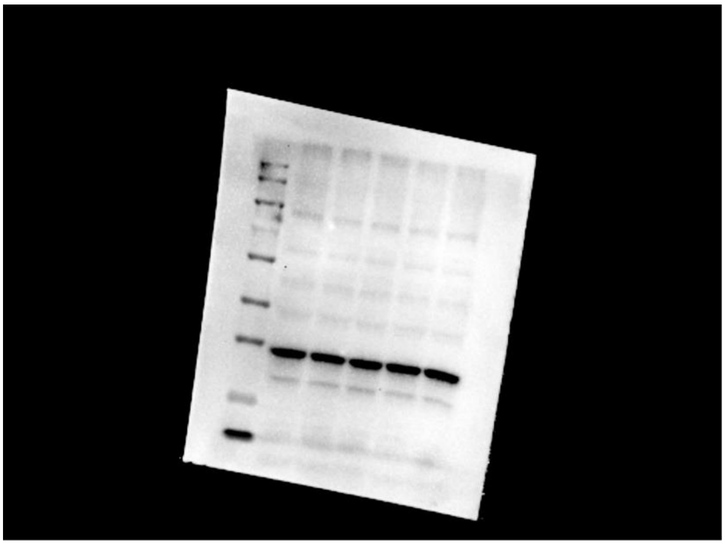
Full and uncropped western blots for Figure 2A-6

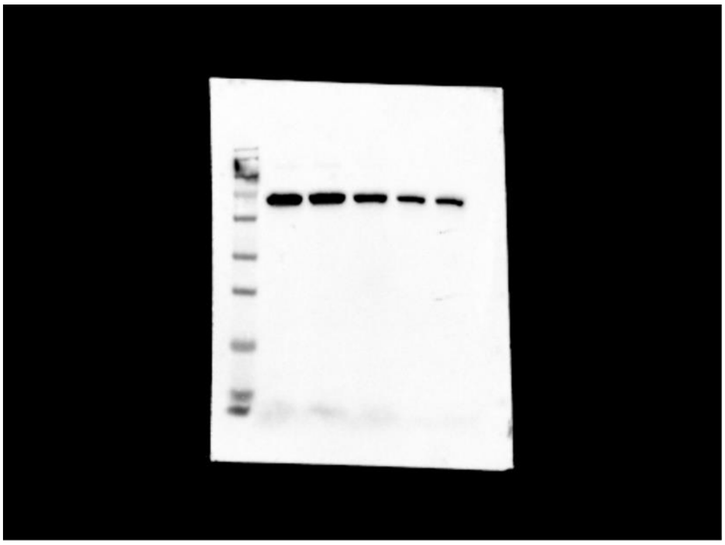

Full and uncropped western blots for Figure 2C-1

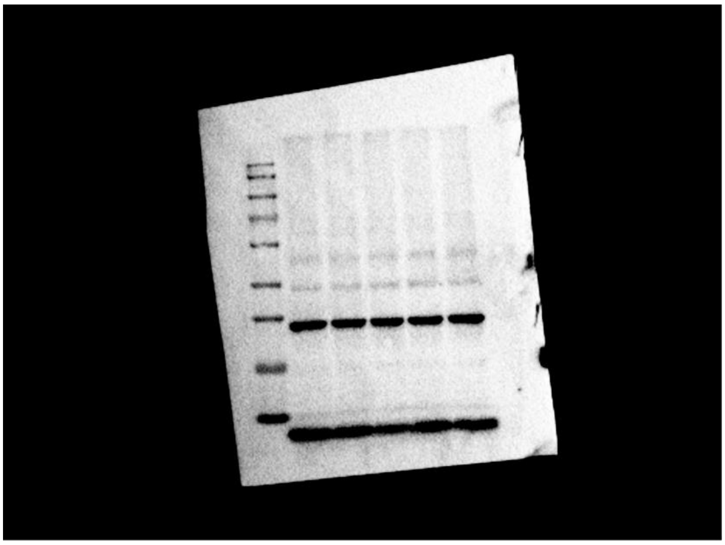

Full and uncropped western blots for Figure 2C-2

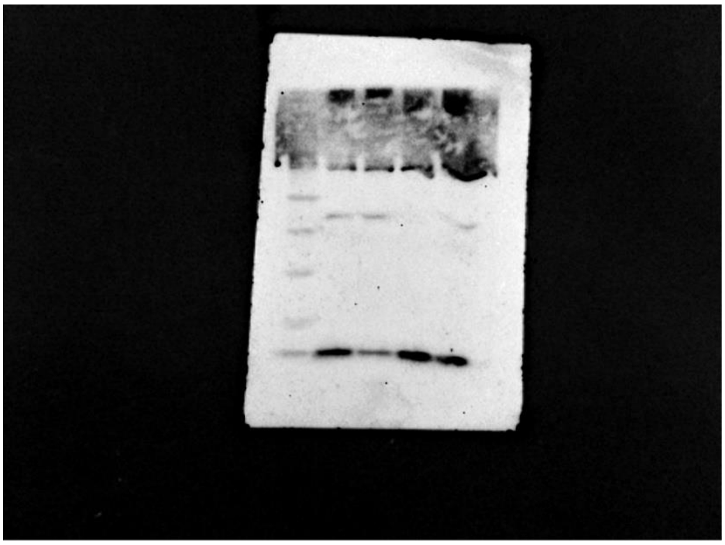

Full and uncropped western blots for Figure 2G-1

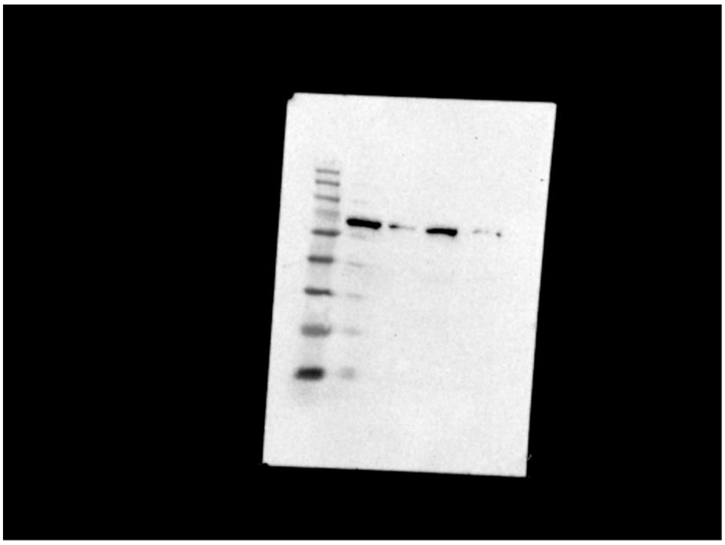

Full and uncropped western blots for Figure 2G-2

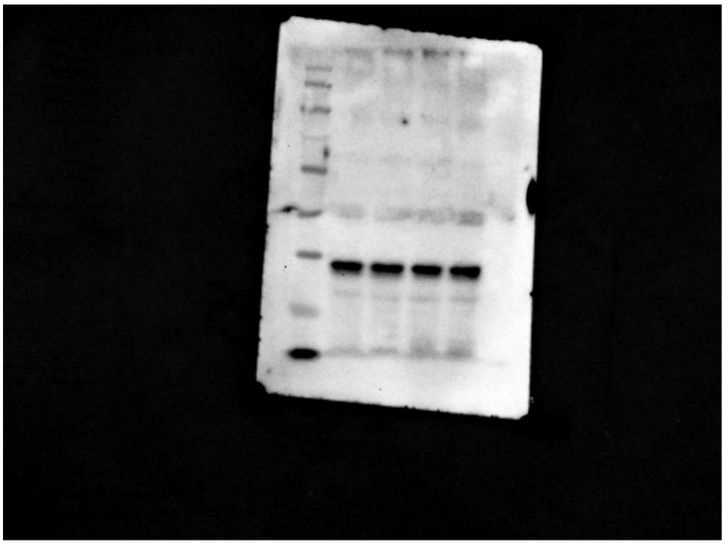

Full and uncropped western blots for Figure 2G-3

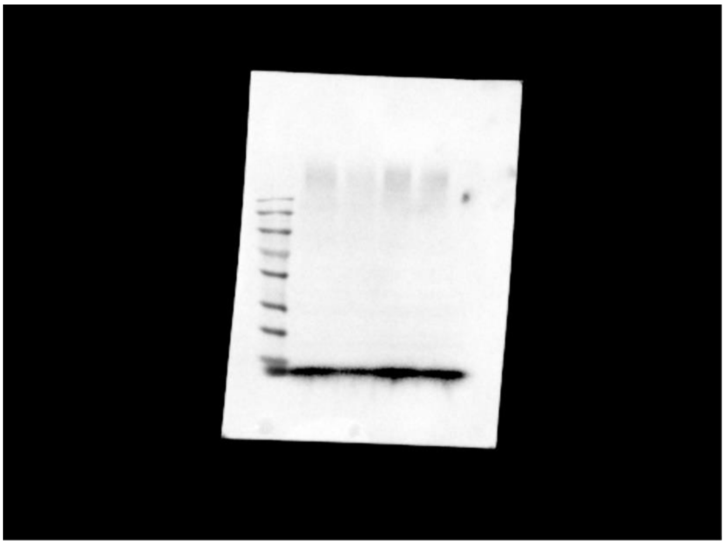

Full and uncropped western blots for Figure 2M-1

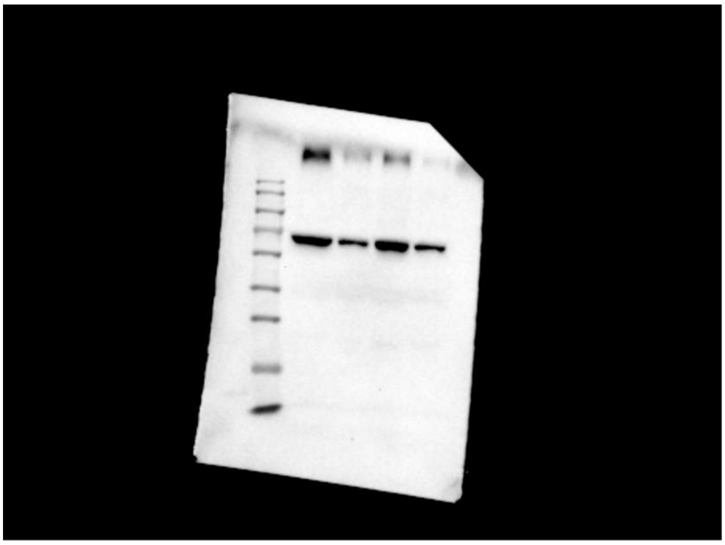

Full and uncropped western blots for Figure 2M-2

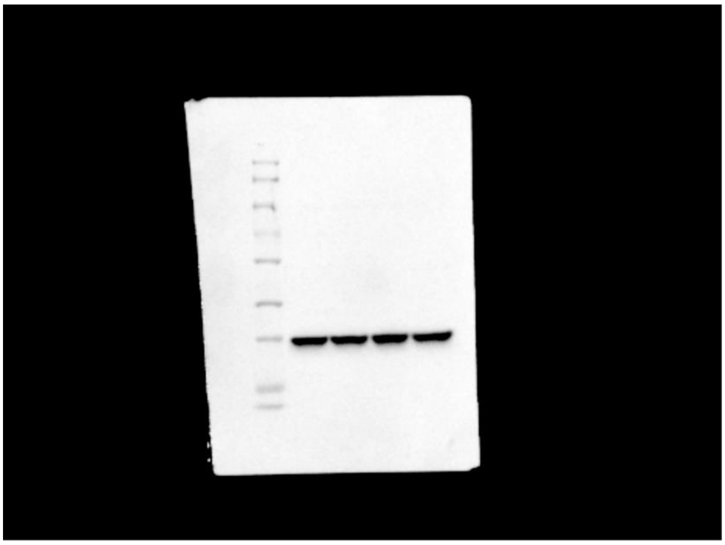

Full and uncropped western blots for Figure 2M-3

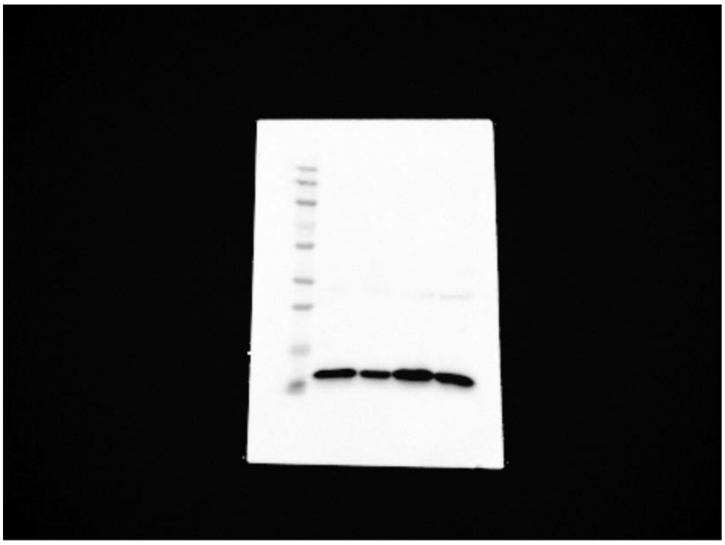

Full and uncropped western blots for Figure 3C-1

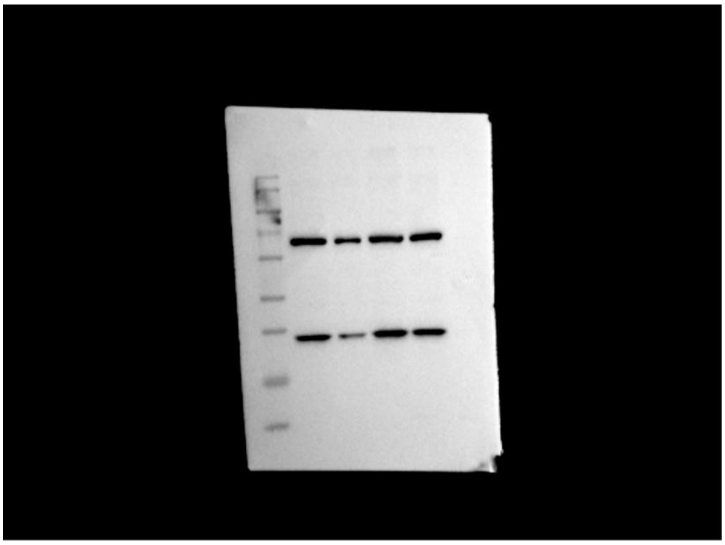
Full and uncropped western blots for Figure 3C-2

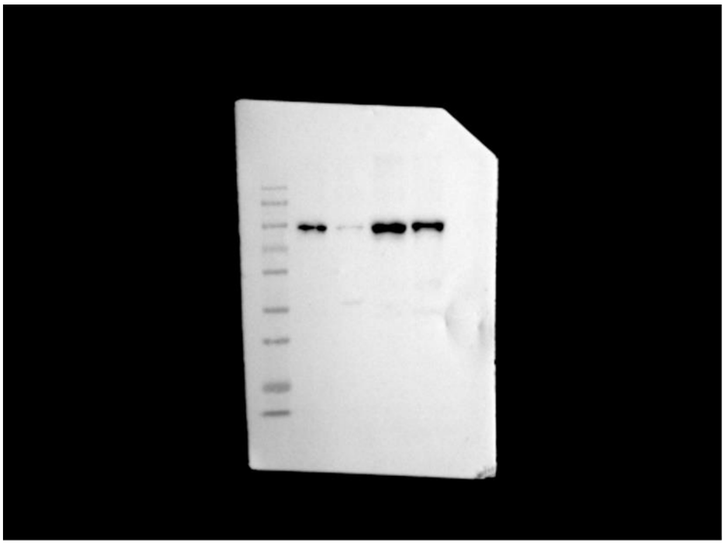
Full and uncropped western blots for Figure 3C-3

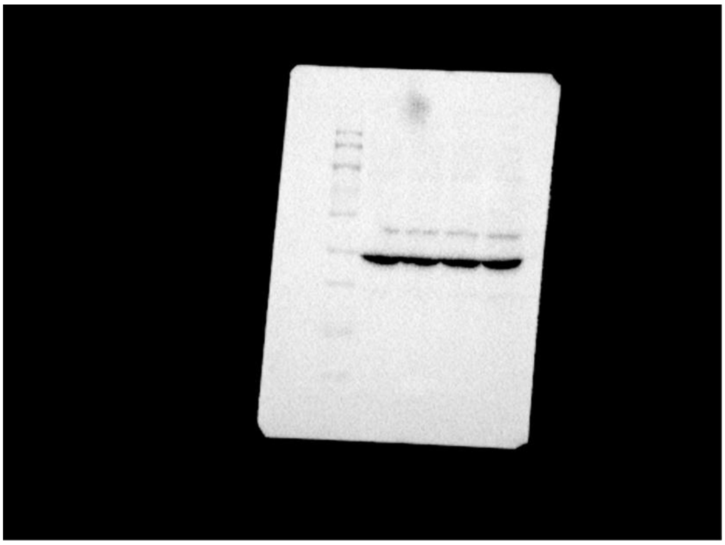
Full and uncropped western blots for Figure 3C-4

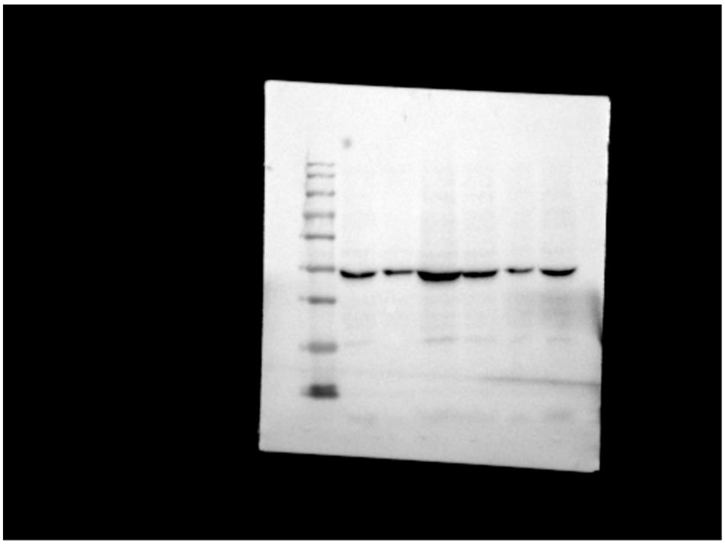

Full and uncropped western blots for Figure 4B-1

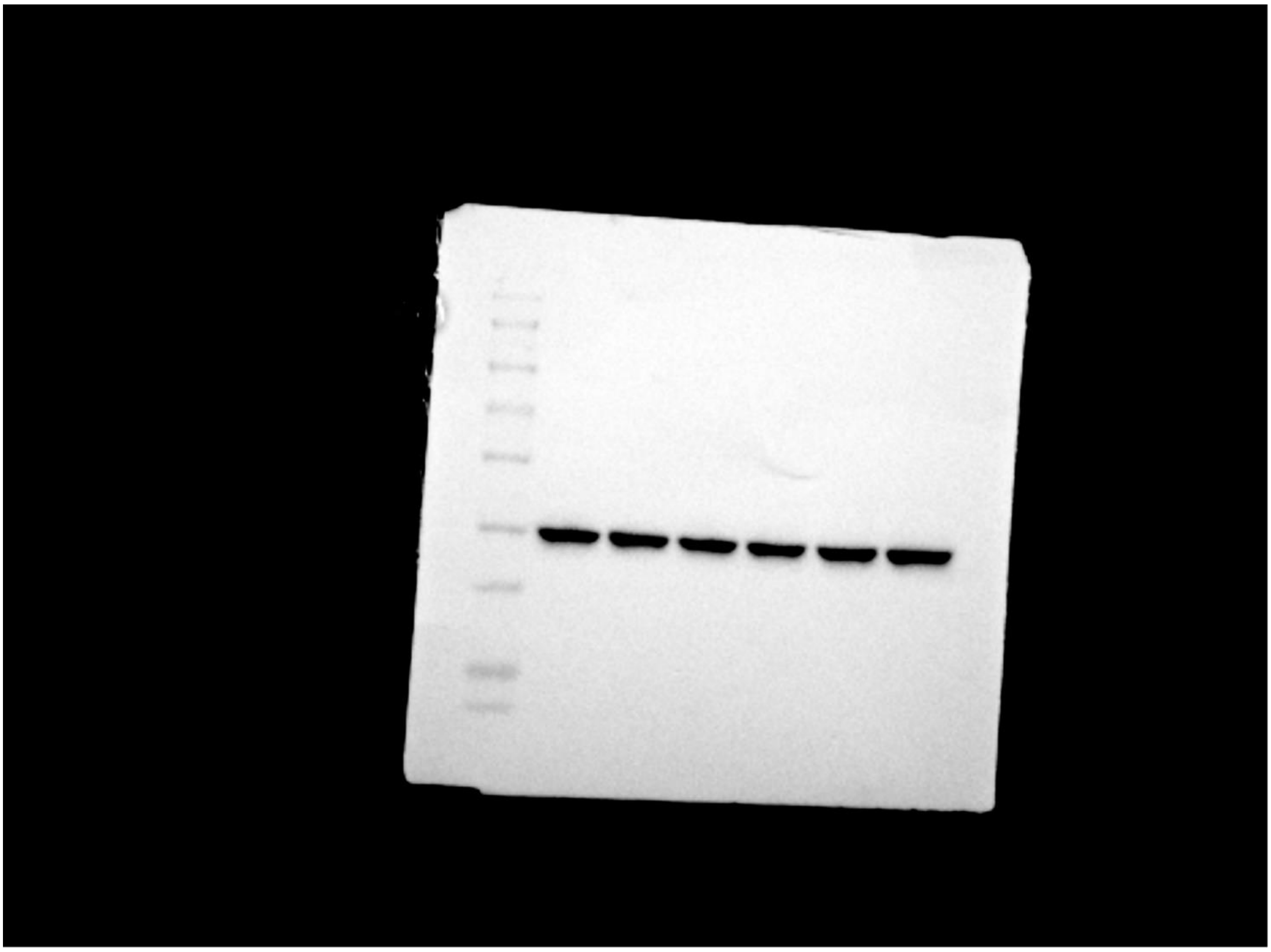

Full and uncropped western blots for Figure 4B-2

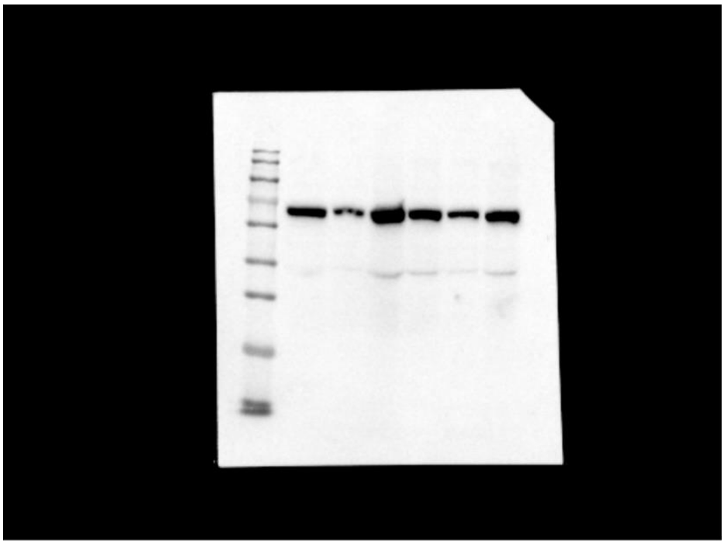

Full and uncropped western blots for Figure 4B-3

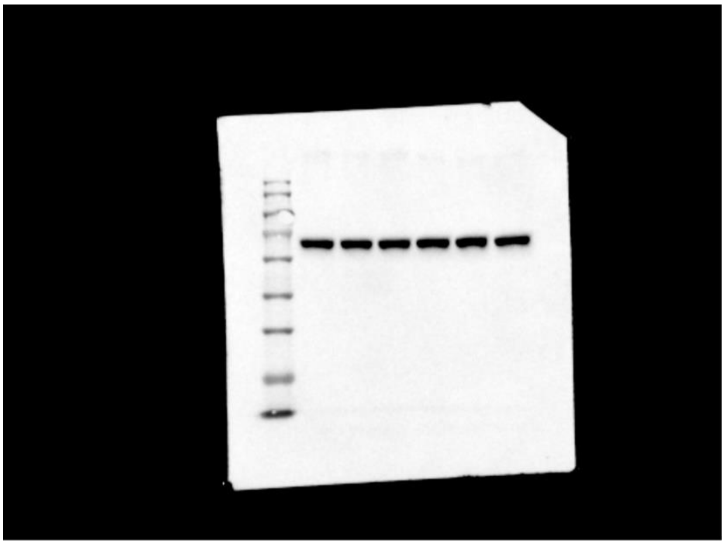

Full and uncropped western blots for Figure 4B-4

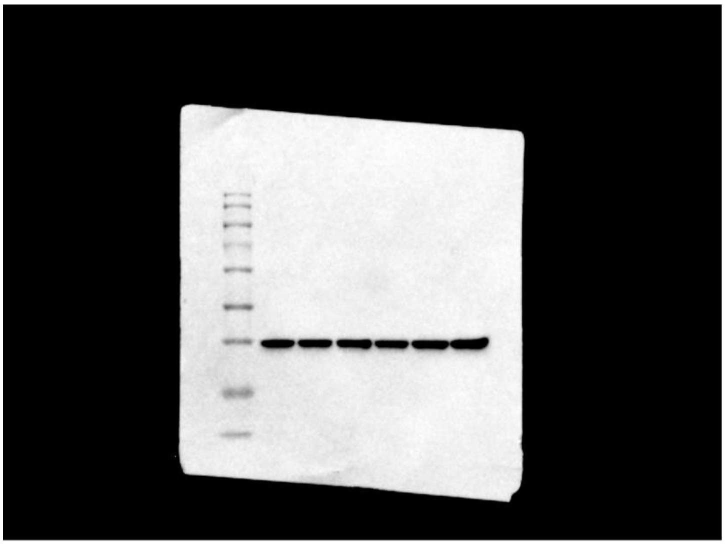

Full and uncropped western blots for Figure 4B-5

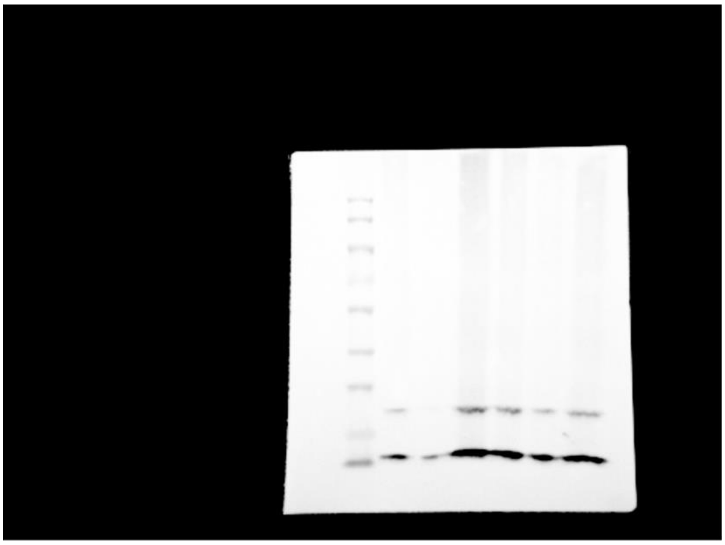

Full and uncropped western blots for Figure 5C-1

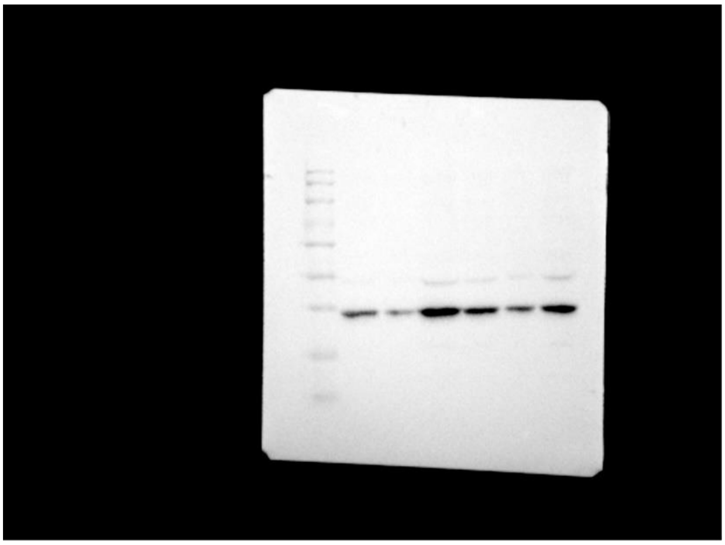

Full and uncropped western blots for Figure 5C-2

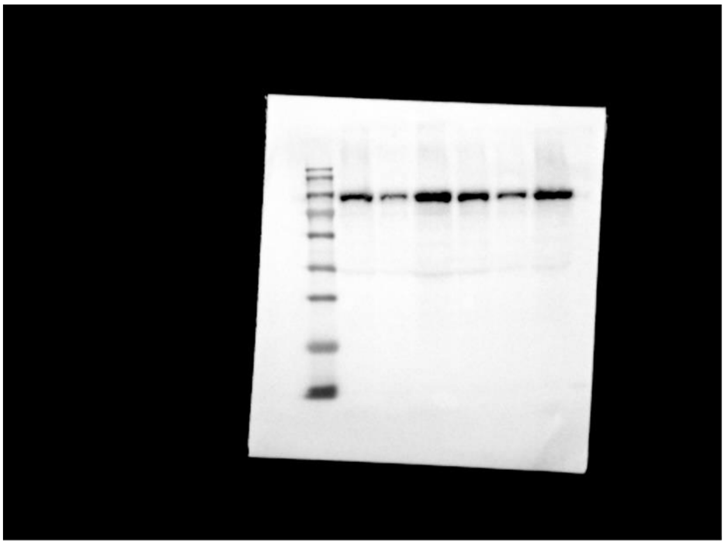

Full and uncropped western blots for Figure 5C-3

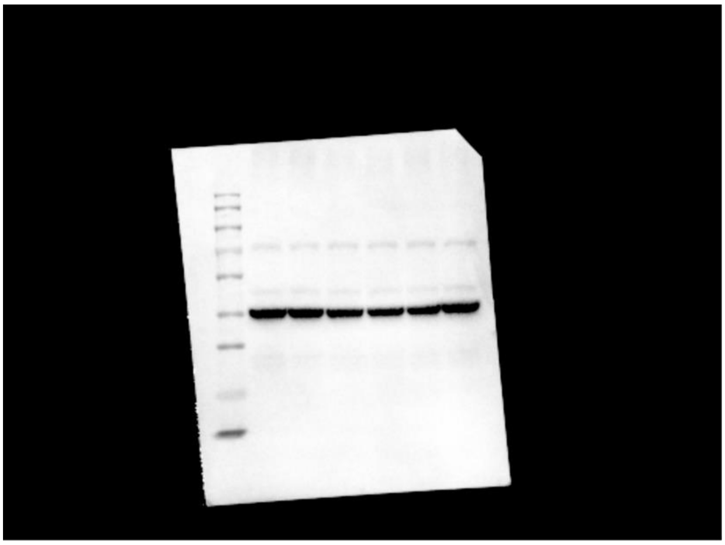

Full and uncropped western blots for Figure 5C-4

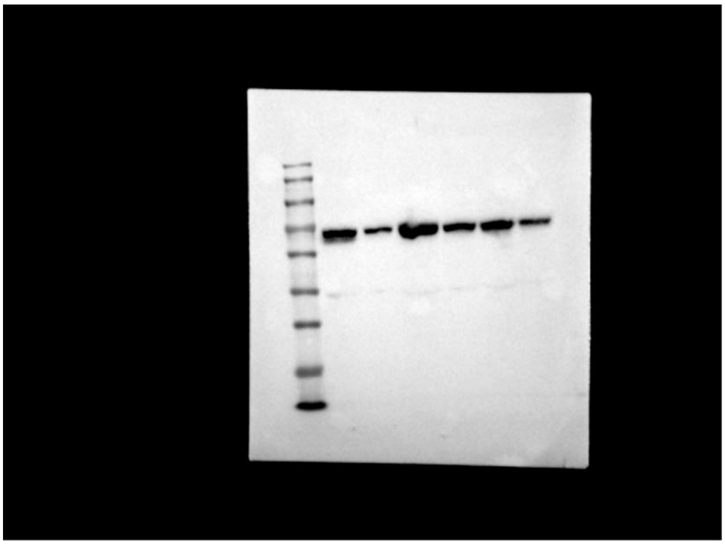

Full and uncropped western blots for Figure 6A-1 (1)

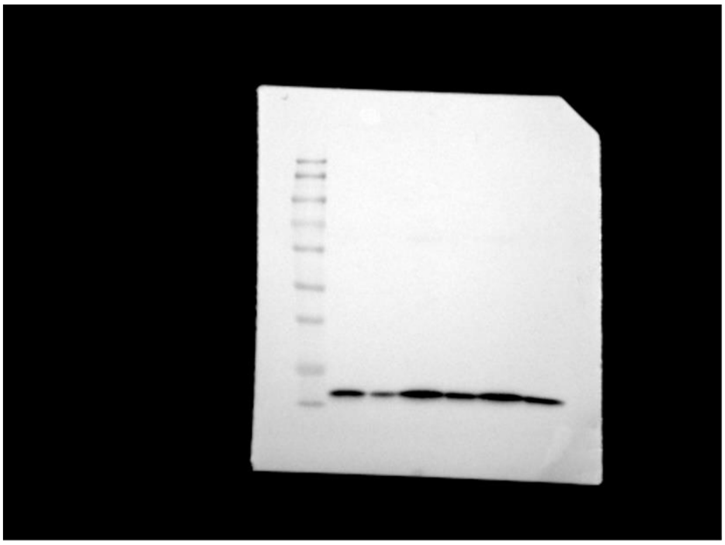

Full and uncropped western blots for Figure 6A-1 (2)

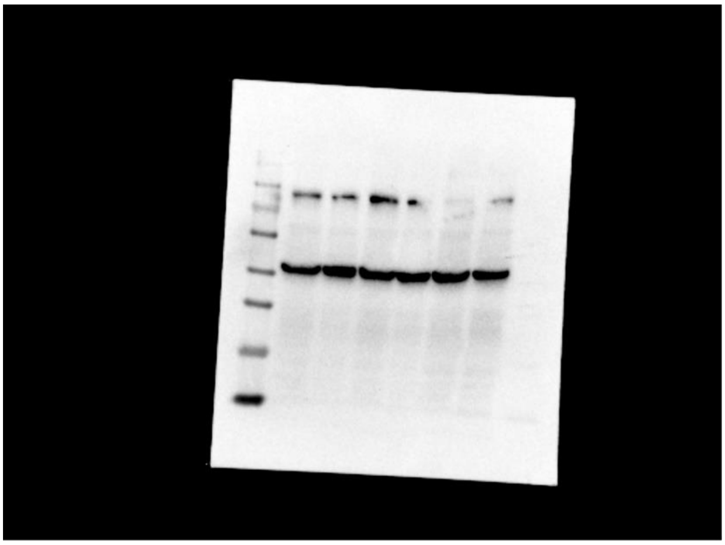

Full and uncropped western blots for Figure 6A-1 (4)

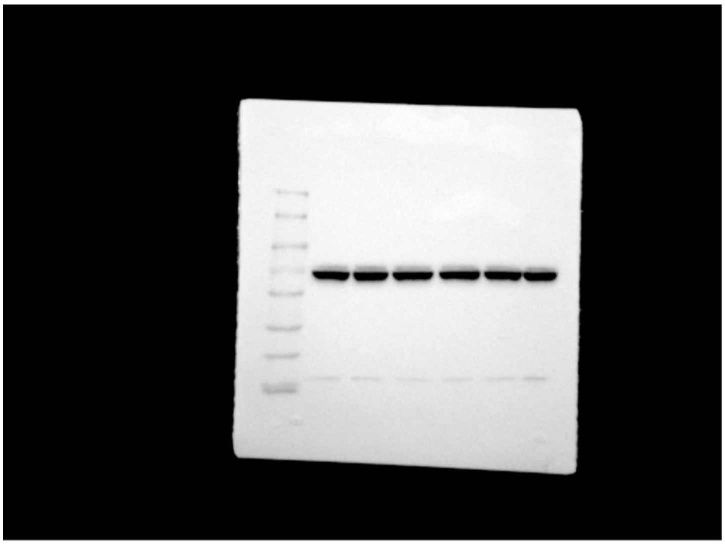

Full and uncropped western blots for Figure 6A-1 (6)

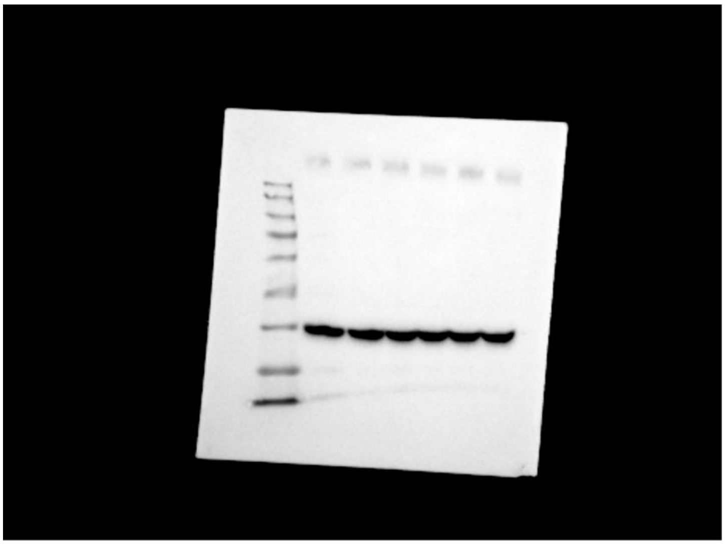

Full and uncropped western blots for Figure 6A-1 (7)

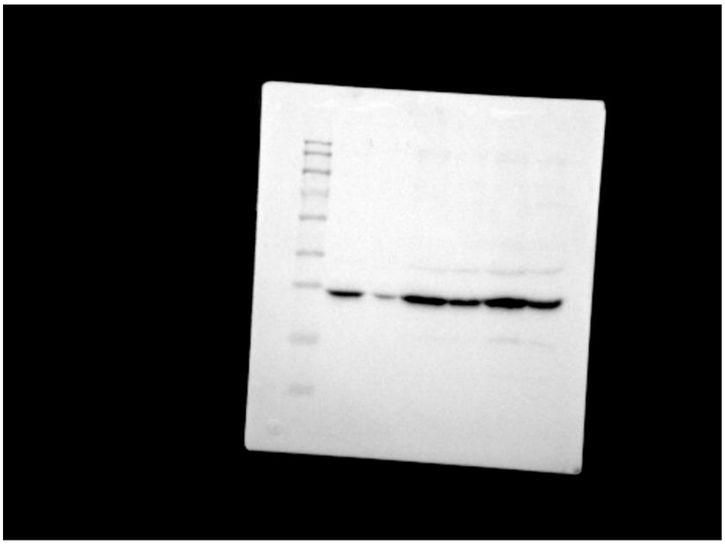

Full and uncropped western blots for Figure 6A-3

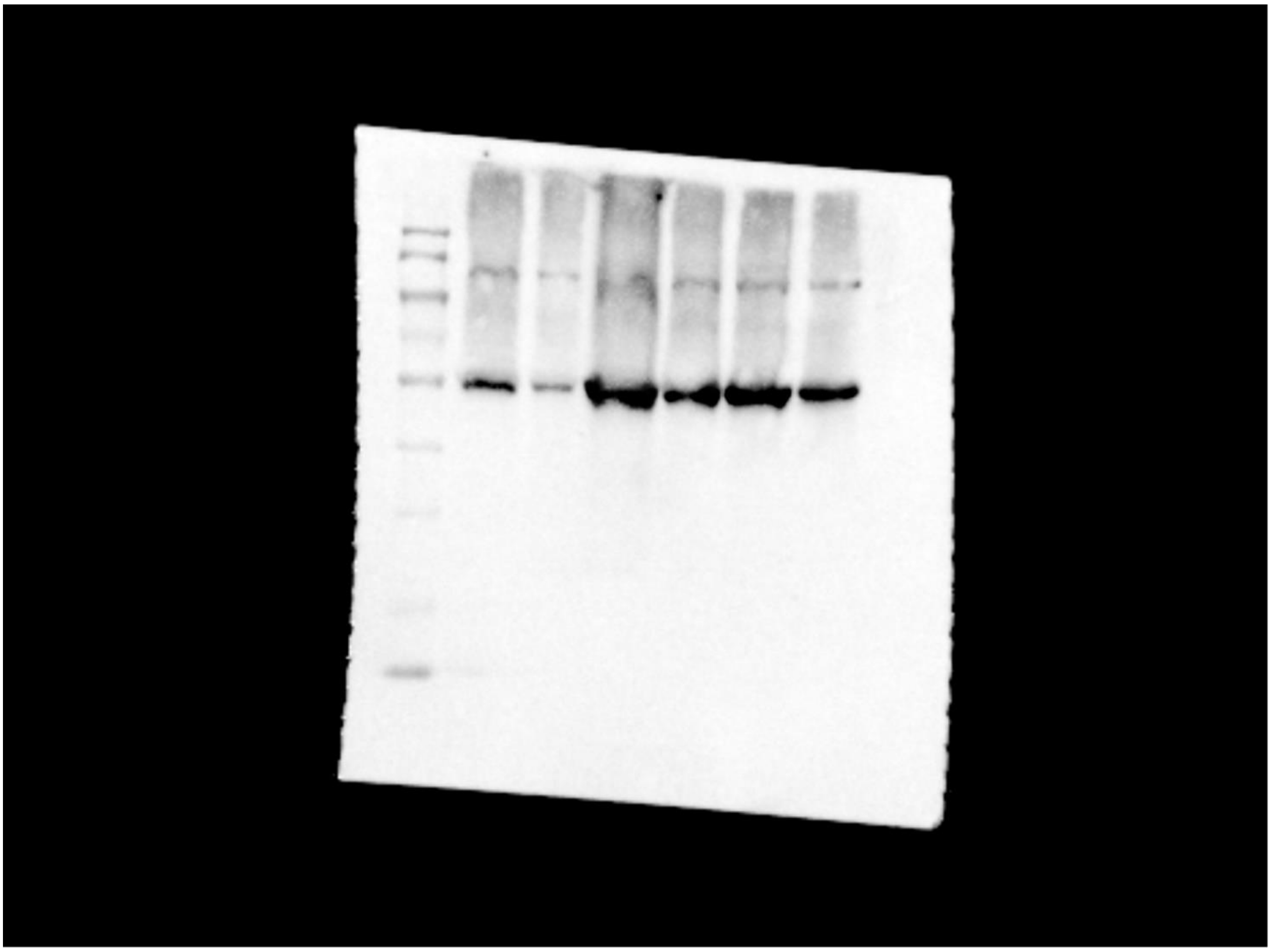

Full and uncropped western blots for Figure 6A-5

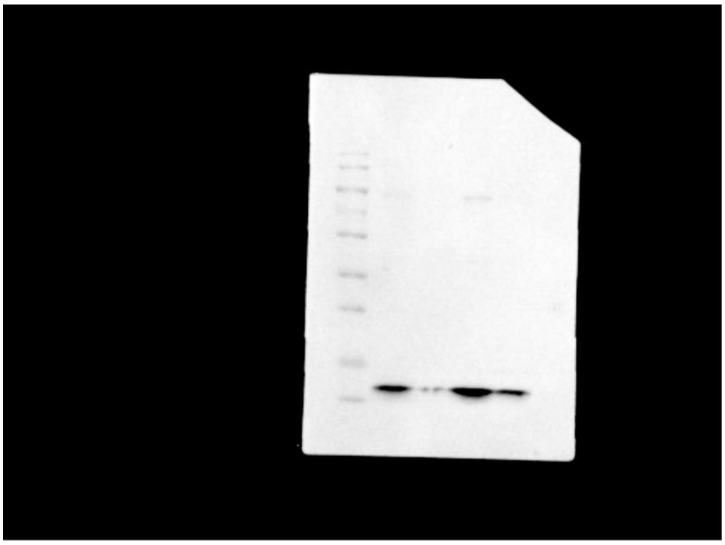

Full and uncropped western blots for Figure 7C-1 (1)

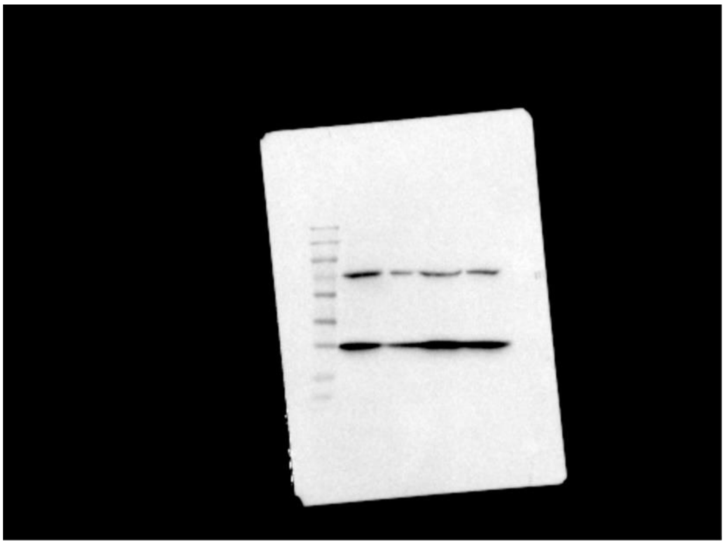

Full and uncropped western blots for Figure 7C-1 (2)

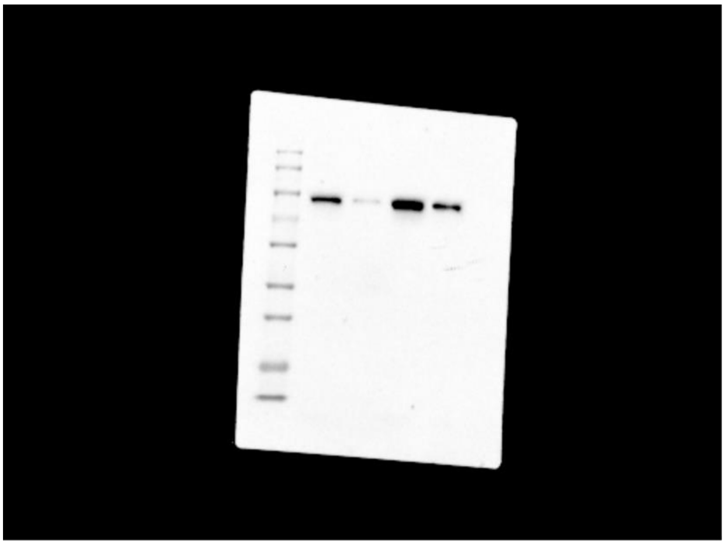

Full and uncropped western blots for Figure 7C-1 (3)

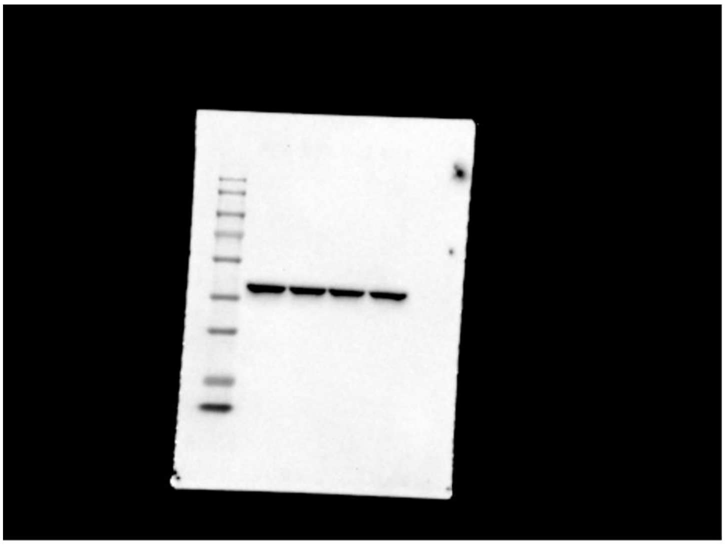

Full and uncropped western blots for Figure 7C-1 (4)

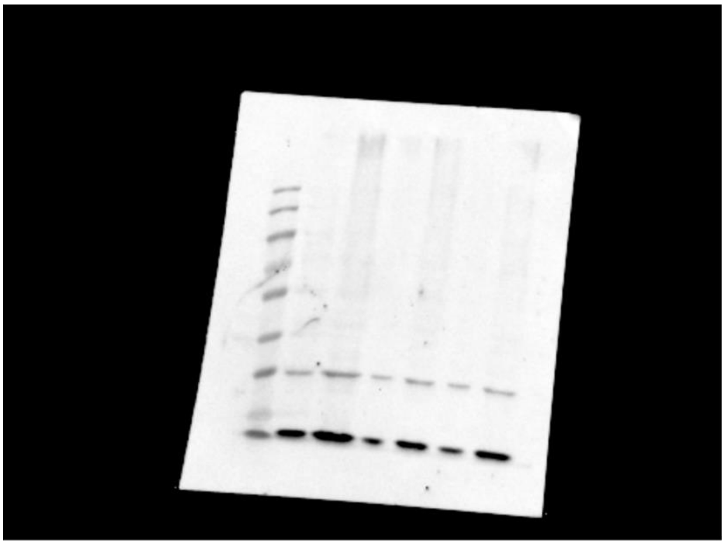

Full and uncropped western blots for Figure 8E-1

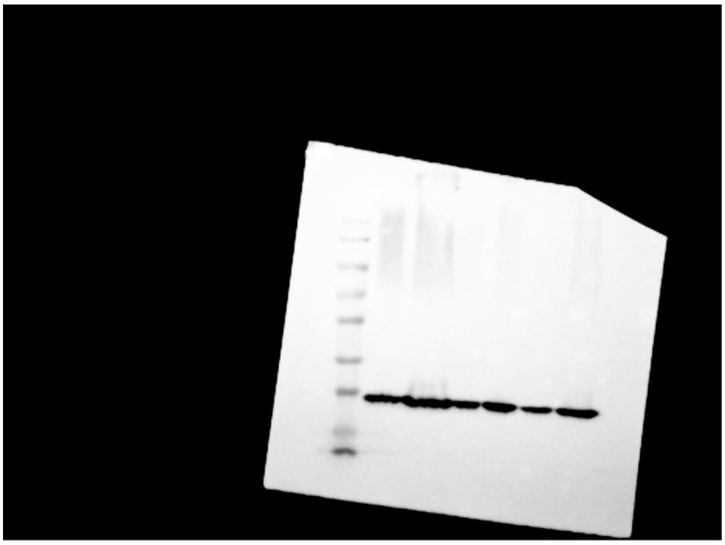

Full and uncropped western blots for Figure 8E-2

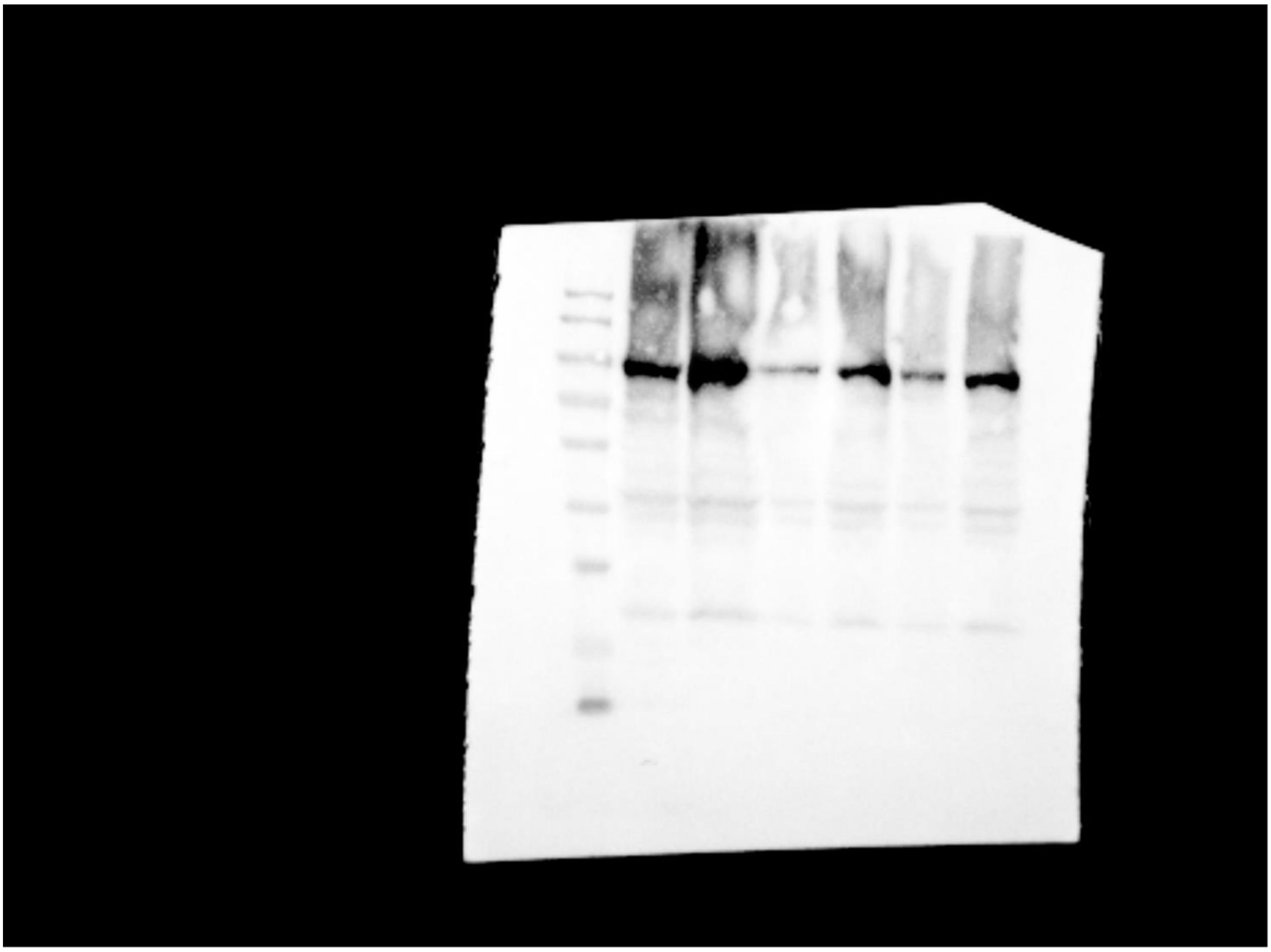

Full and uncropped western blots for Figure 8E-3

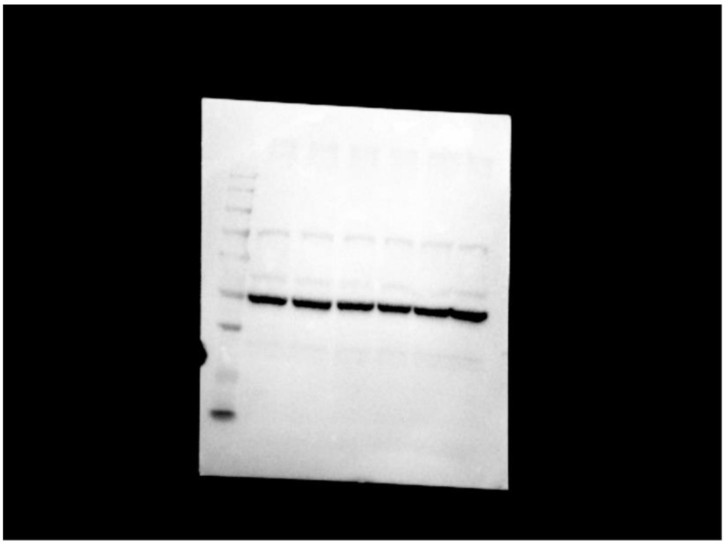

Full and uncropped western blots for Figure 8E-4

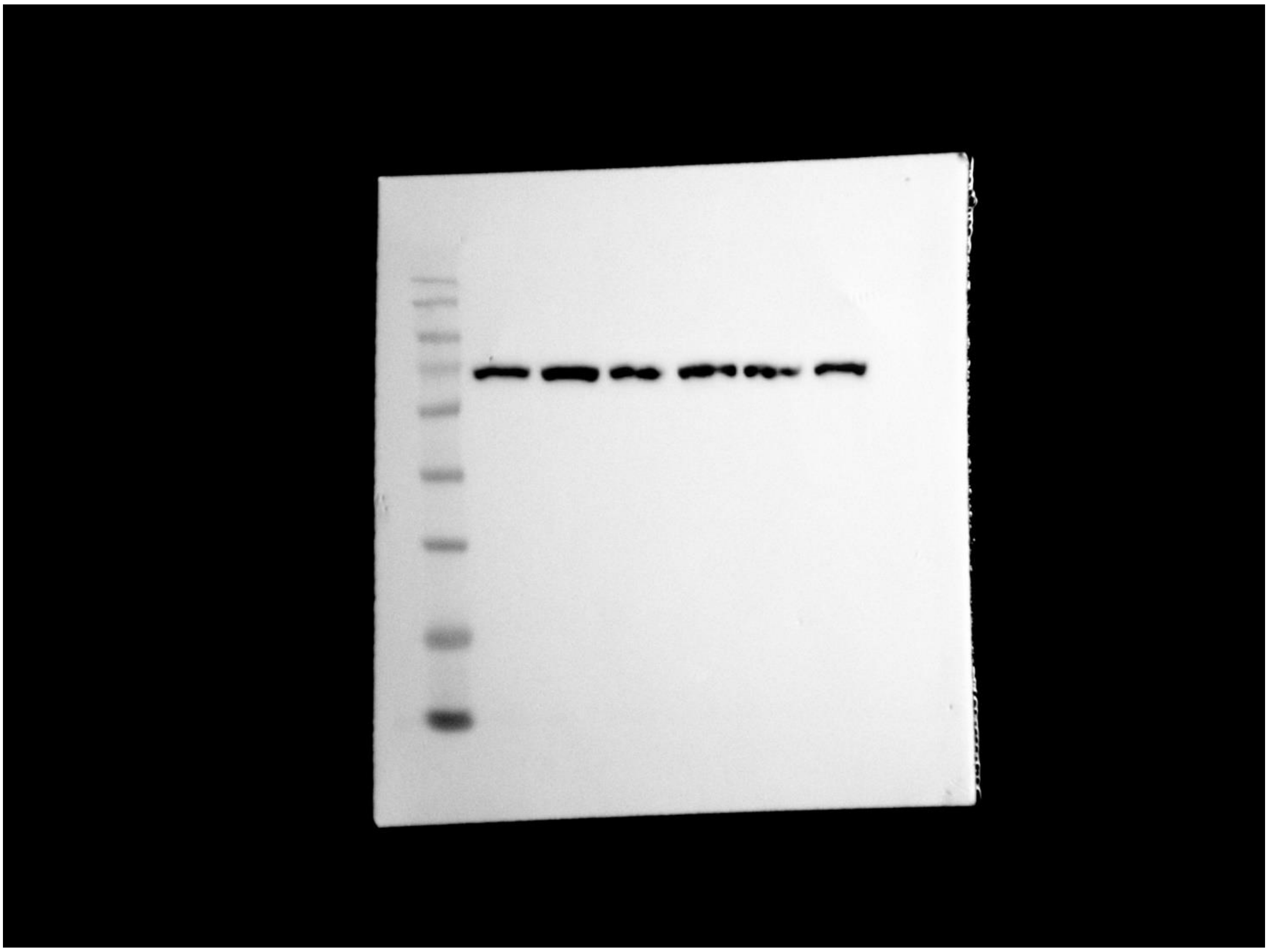
Full and uncropped western blots for Figure 9A-1 (1)

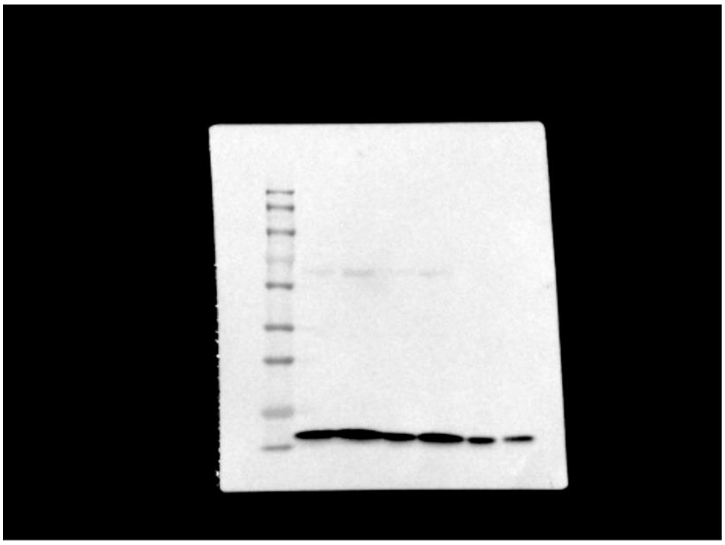
Full and uncropped western blots for Figure 9A-1 (2)

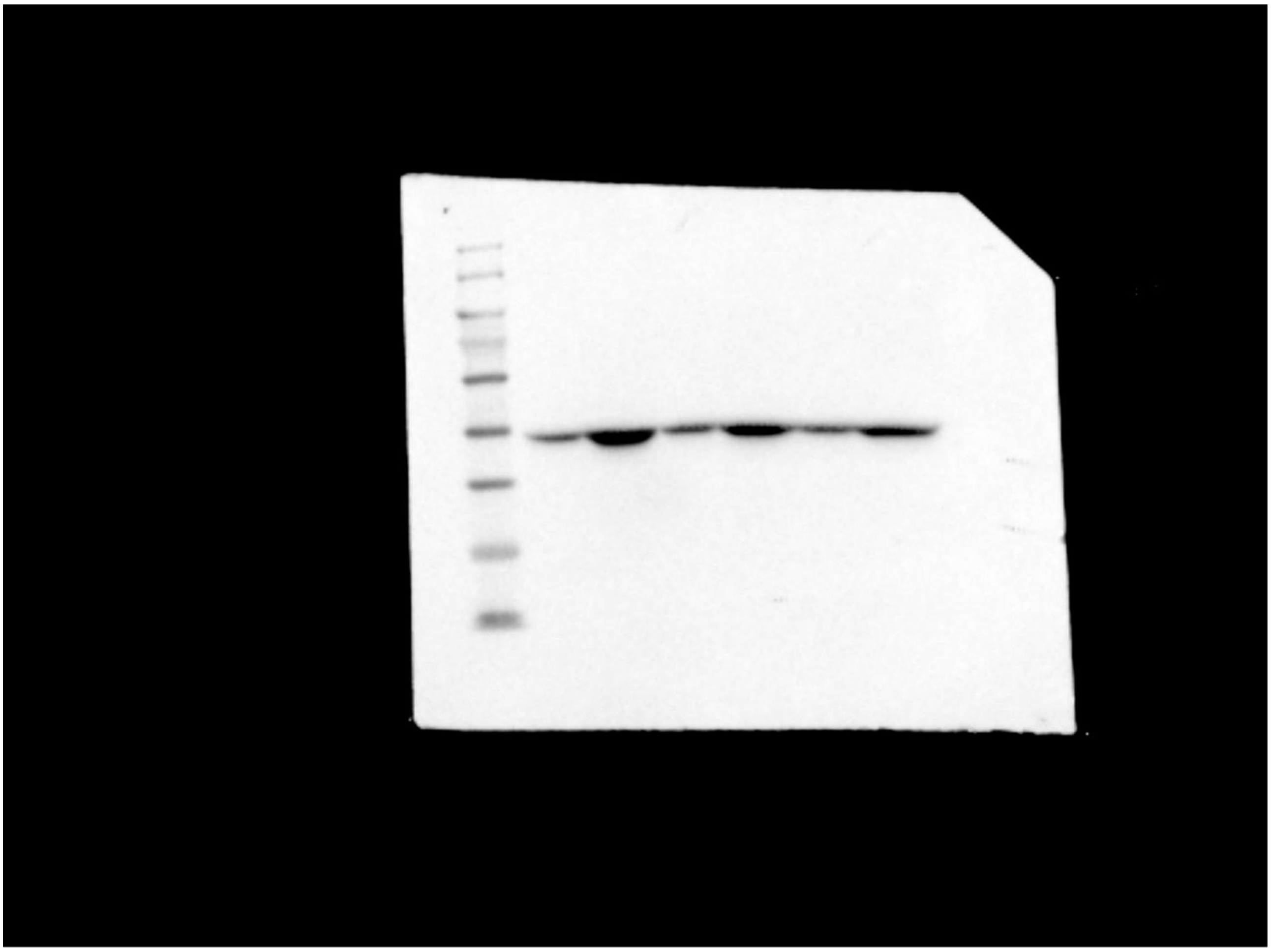

Full and uncropped western blots for Figure 9A-1 (3)

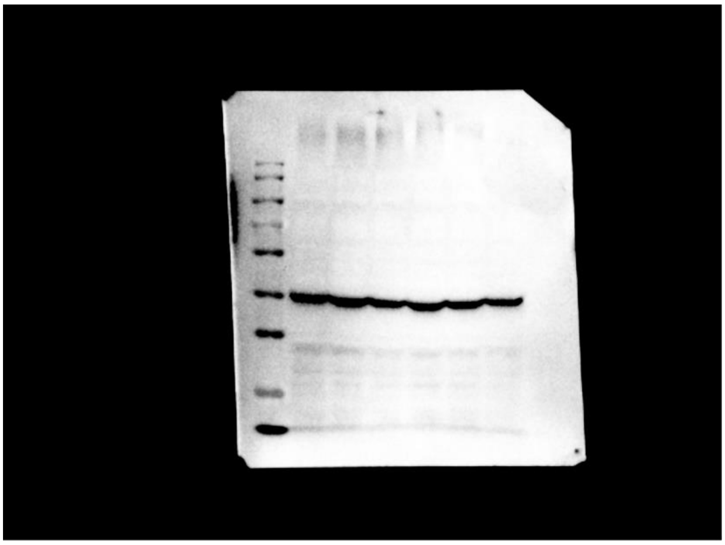

Full and uncropped western blots for Figure 9A-1 (4)

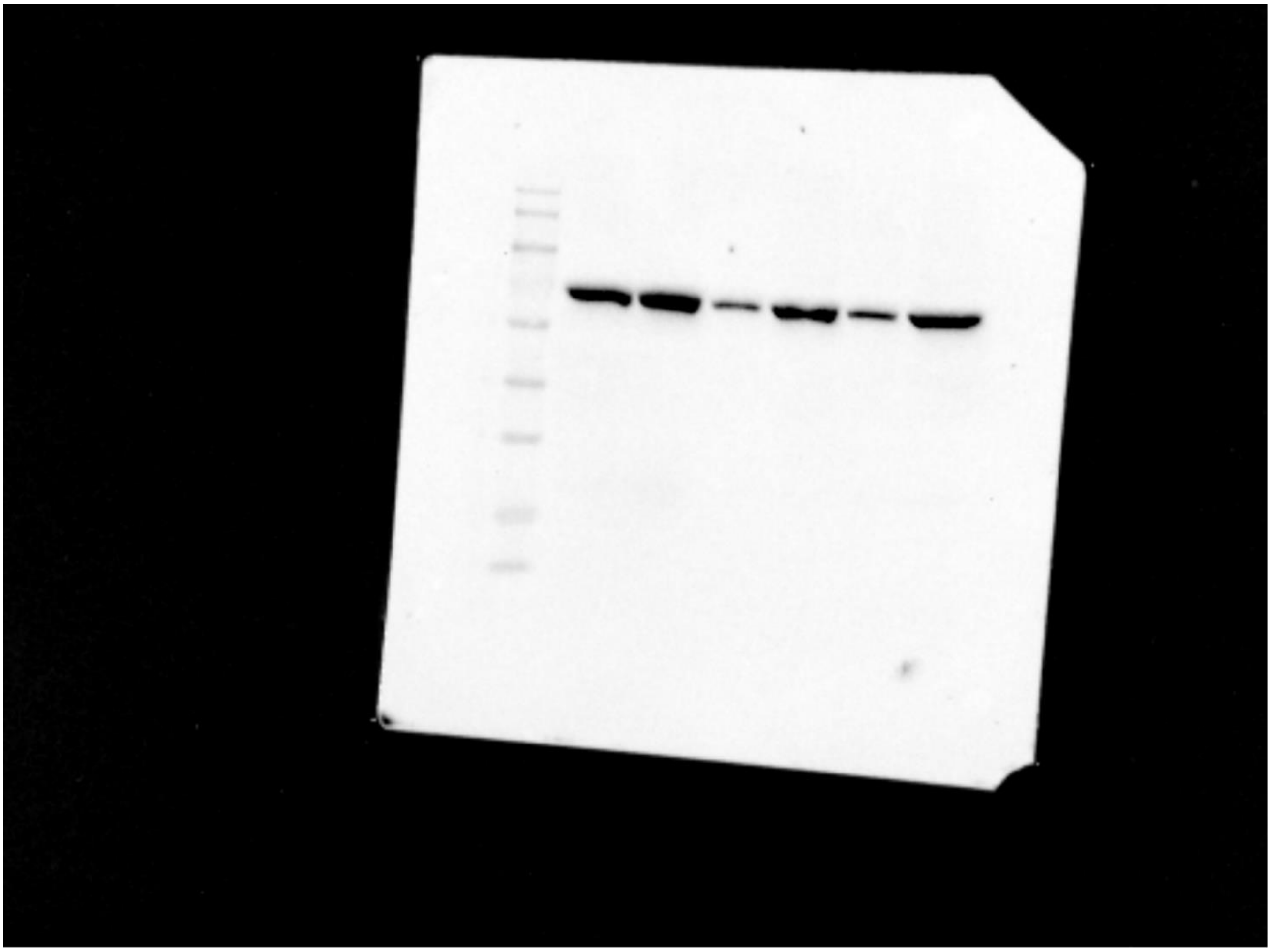
Full and uncropped western blots for Figure 9A-1 (5)

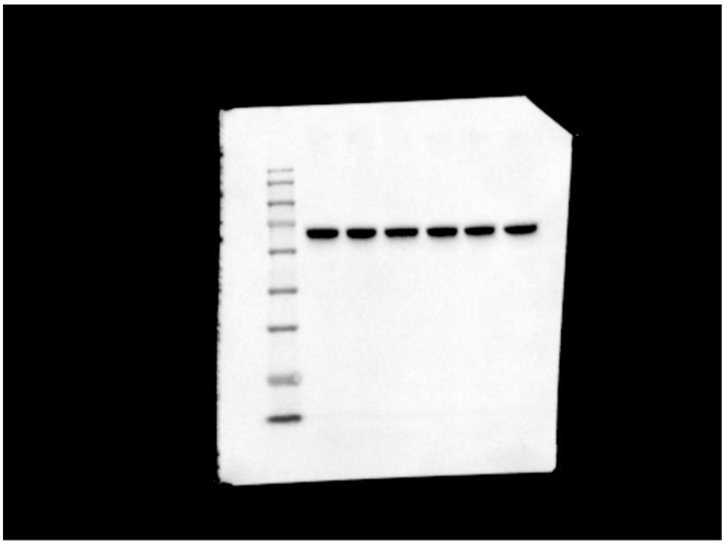
Full and uncropped western blots for Figure 9A-1 (6)

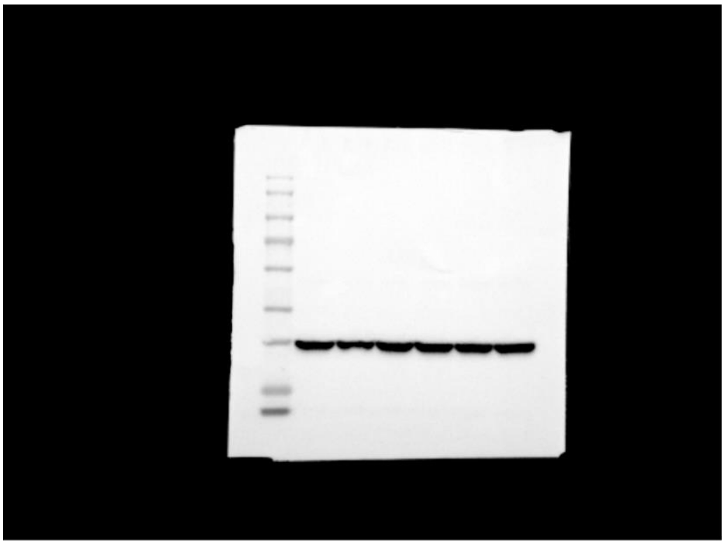
Full and uncropped western blots for Figure 9A-1 (7)
